# Building a Digital Bridge to Support Patient-Centered Care Transitions From Hospital to Home for Older Adults With Complex Care Needs: Protocol for a Co-Design, Implementation, and Evaluation Study

**DOI:** 10.2196/20220

**Published:** 2020-11-25

**Authors:** Carolyn Steele Gray, Terence Tang, Alana Armas, Mira Backo-Shannon, Sarah Harvey, Kerry Kuluski, Mayura Loganathan, Jason X Nie, John Petrie, Tim Ramsay, Robert Reid, Kednapa Thavorn, Ross Upshur, Walter P Wodchis, Michelle Nelson

**Affiliations:** 1 Bridgepoint Collaboratory for Research and Innovation Lunenfeld-Tanenbaum Research Institute Sinai Health System Toronto, ON Canada; 2 Institute for Health Policy, Management and Evaluation Dalla Lana School of Public Health University of Toronto Toronto, ON Canada; 3 Institute for Better Health Trillium Health Partners Toronto, ON Canada; 4 Department of Family and Community Medicine University of Toronto Toronto, ON Canada; 5 Clinical, Health System Strategy, Integration and Planning Ontario Health (Central Region) Mississauga Halton Local Health Integration Network Toronto, ON Canada; 6 QoC Health Inc Toronto, ON Canada; 7 Mount Sinai Academic Family Health Team Toronto, ON Canada; 8 Ottawa Methods Centre Ottawa Hospital Research Institute The Ottawa Hospital Ottawa, ON Canada; 9 School of Epidemiology Public Health and Preventative Medicine University of Ottawa Ottawa, ON Canada; 10 March of Dimes Canada Toronto, ON Canada

**Keywords:** digital health technology, care transitions, multimorbidity, pragmatic trial, co-design, hospital, primary care

## Abstract

**Background:**

Older adults with multimorbidity and complex care needs (CCN) are among those most likely to experience frequent care transitions between settings, particularly from hospital to home. Transition periods mark vulnerable moments in care for individuals with CCN. Poor communication and incomplete information transfer between clinicians and organizations involved in the transition from hospital to home can impede access to needed support and resources. Establishing digitally supported communication that enables person-centered care and supported self-management may offer significant advantages as we support older adults with CCN transitioning from hospital to home.

**Objective:**

This protocol outlines the plan for the development, implementation, and evaluation of a Digital Bridge co-designed to support person-centered health care transitions for older adults with CCN. The Digital Bridge builds on the foundation of two validated technologies: Care Connector, designed to improve interprofessional communication in hospital, and the electronic Patient-Reported Outcomes (ePRO) tool, designed to support goal-oriented care planning and self-management in primary care settings. This project poses three overarching research questions that focus on adapting the technology to local contexts, evaluating the impact of the Digital Bridge in relation to the quadruple aim, and exploring the potential to scale and spread the technology.

**Methods:**

The study includes two phases: workflow co-design (phase 1), followed by implementation and evaluation (phase 2). Phase 1 will include iterative co-design working groups with patients, caregivers, hospital providers, and primary care providers to develop a transition workflow that will leverage the use of Care Connector and ePRO to support communication through the transition process. Phase 2 will include implementation and evaluation of the Digital Bridge within two hospital systems in Ontario in acute and rehab settings (600 patients: 300 baseline and 300 implementation). The primary outcome measure for this study is the Care Transitions Measure–3 to assess transition quality. An embedded ethnography will be included to capture context and process data to inform the implementation assessment and development of a scale and spread strategy. An Integrated Knowledge Translation approach is taken to inform the study. An advisory group will be established to provide insight and feedback regarding the project design and implementation, leading the development of the project knowledge translation strategy and associated outputs.

**Results:**

This project is underway and expected to be complete by Spring 2024.

**Conclusions:**

Given the real-world implementation of Digital Bridge, practice changes in the research sites and variable adherence to the implementation protocols are likely. Capturing and understanding these considerations through a mixed-methods approach will help identify the range of factors that may influence study results. Should a favorable evaluation suggest wide adoption of the proposed intervention, this project could lead to positive impact at patient, clinician, organizational, and health system levels.

**Trial Registration:**

ClinicalTrials.gov NCT04287192; https://clinicaltrials.gov/ct2/show/NCT04287192

**International Registered Report Identifier (IRRID):**

PRR1-10.2196/20220

## Introduction

### Background

Older adults with multimorbidity and complex care needs (CCN) are among those most likely to experience frequent care transitions between settings, particularly from hospital to home [1,2]. Many of these community-dwelling older adults fall into the category of high-cost users [3], who account for the majority of year over year health care spending internationally [4-7]. The complexity of these individuals stems not only from their multimorbidity disease profiles but also the social, environmental, and contextual issues that make it difficult for them to manage their physical and mental health needs [8]. It is often the interaction of these challenges that results in frequent visits to the hospital.

When patients leave the hospital, they face challenges as they attempt to cope and adjust at home. Krumholz [9] coined the term posthospital syndrome to describe this acquired, transient period of vulnerability post-discharge due to impaired physiological systems and depleted reserves. This depletion limits patients’ ability to adjust and manage their health issues, often leading to hospital readmission within 30 days with an acute medical illness unrelated to the original diagnosis. Poor communication and incomplete information transfer between the various clinicians and organizations providing care to CCN patients as they transition from hospital to home can lead to medication errors, readmissions, decreased patient satisfaction, further morbidity, and even mortality [10]. These issues can be exacerbated in smaller communities where resources and services may be lacking, transportation limitations may exist, providers may be limited, or wait times may be increased [11,12]. Studies have demonstrated that insufficient communication during the transition process can lead to poor patient outcomes and higher rates of readmission for older adults with CCN [13,14].

While improving clinician communication is important, the quality and content of that communication with patients also matters. Patients with CCN benefit most from person-centered delivery models that can adapt to their unique needs and engage them as partners in their care [15,16]. Person-centered approaches have been shown to improve discharge from hospital to home by emphasizing partnership between patient and provider, improving patient self-efficacy, and improving communication between patients and providers and within care teams [17-19]. For patients with CCN, incorporating ongoing support for self-care after they return home as part of that communication can offer additional support and benefit [20]. In sum, communication that enables person-centered care and supported self-management may offer the greatest advantages as we support older adults with CCN transitioning from hospital to home.

Digital health technologies offer a promising solution to support person-centered communication across interprofessional teams working within and across health care organizations [21-26]. A systematic review of interprofessional communication in transitional care models found that information systems and multiprofessional care coordination support higher satisfaction and subjective quality of life for older adults [27]. A key strength of digital solutions is their ability to potentially foster shared situational awareness to support clinical decision making across care teams [28,29] of interprofessional teams. An essential component of interdisciplinary communication [30], shared situational awareness is a group or team’s ability to understand the big picture and work together toward a common goal [31,32], like transitioning a patient from hospital to home. Digital solutions have the ability to both synchronously and asynchronously enable information sharing regarding a team’s common goal.

While these examples demonstrate the potential of digital communication platforms to improve team communication and functioning, a number of issues remain that limit the value of current systems. First, the majority of communication systems exist within single teams or organizations and rarely span those boundaries [33]. Second, many available communication systems do not inherently support person-centered care delivery, as few are co-designed with patients and providers [34]. Third, digital health solutions have been criticized for limiting other forms of information communication needed to foster shared situational awareness [35]. As such, many of the available systems are not well suited to supporting the communication needs of care teams, patients, and families during the time of transition from hospital back to the community. Finally, many existing systems have only been evaluated over short periods with insufficient attention to implementation as a means to support both evidence of effectiveness and transferability of findings [36].

Our project will address these gaps by implementing and evaluating a Digital Bridge to support person-centered health care transitions for older adults with CCN. The Digital Bridge will (1) span organizational and professional boundaries by enabling communication between interdisciplinary teams working in hospital and primary care with patients and caregivers, (2) support person-centered delivery through adoption of co-design methods to establish a workflow, and (3) be evaluated through an implementation science lens.

The Digital Bridge will integrate two previously and separately tested and validated technologies that are currently in use in hospital and community settings: (1) Care Connector and (2) the electronic Patient-Reported Outcomes (ePRO) tool. Care Connector is an interprofessional communication and collaboration platform initially designed in the hospital setting to support clinical teams caring for patients with CCN [37]. The tool includes discharge communication supports like Patient-Oriented Discharge Summaries (PODS) [38] to support clinician communication and collaboration in the community and across care settings. The ePRO tool is a primary care–facing technology co-designed with patients with CCN, their primary care providers, and family caregivers to enable communication of patient-oriented goals [39].

While ePRO and Care Connector have been designed and tested in their single settings (primary care and hospital, respectively), bringing the solutions together to create the Digital Bridge will build in new functionality and workflows that are, as yet, untested. This protocol outlines a development (co-designing the integration of the solutions), implementation (putting the new Digital Bridge into practice), and evaluation (testing impact of the Digital Bridge) study. We hypothesize that these two technologies will work synergistically by supporting communication and collaboration needs of clinicians and patients at the critical time of care transitions (Care Connector) and engaging patients to set goals and monitor progress throughout transitions from hospital to living in the community over the longer term (ePRO).

### Objective and Research Questions

This project poses three overarching research questions aimed at adapting the technology to local contexts (RQ1), evaluating the impact of the Digital Bridge (RQ2), and exploring the potential to scale and spread the technology (RQ3).

1. What are the workflow design considerations in adopting digital solutions that bridge care settings to support transitions from hospital to home for patients with CCN, from patient/caregiver, clinician, and organizational perspectives?
2. Does the digital solution achieve quadruple aim goals by offering a cost-effective means of supporting care transitions to achieve improved provider experience (improved communication around transitions and improved teaming), patient experience with transitions (improved person-centered care transitions), and patient-reported outcomes (health-related quality of life)?
3. What are the implementation enablers and barriers to adopting technology in this process from patient, caregiver, provider, organizational, and system perspectives?

Our proposed project will advance learning in three areas, each of which has strong potential for downstream impacts for patients, providers, and health systems. First, this project will further our understanding of person-centered transition models of care through the co-design of the workflow and evaluation of its impacts. Second, we will be among the first to integrate hospital-based and primary care–based digital health technologies to support care transitions, addressing a critical integration challenge identified by health system decision makers. We will learn how to build stable and secure integrated data architectures, establish productive partnerships across multiple stakeholders, and determine the costs and values of this type of integration. Finally, the project will develop a template for adopting innovative models and digital technologies to support older adults with CCN that cross organizational and professional boundaries. Drawing on implementation science theory to guide our work allows us to produce recommendations on how to adopt similar boundary-spanning technologies in different health care settings.

### Setting and Context

To ensure the designed intervention is potentially scalable, we will carry out this project in two distinct health care organizations in Ontario, Canada. Sinai Health System (SHS) is a hospital system located in Toronto, Ontario, comprising two hospitals (Mount Sinai and Bridgepoint), two family health teams, and a community agency (Circle of Care). The hospitals have a total of 831 beds in-service, 29,062 admissions to Mount Sinai, 19,611 outpatient visits to Bridgepoint, with Circle of Care providing over 1.4 million hours of personal support and rehabilitation to people in their homes [34]. Located in Mississauga, Ontario, Trillium Health Partners (THP) is one of Canada’s largest community-based teaching hospitals, affiliated with the University of Toronto. In 2018-2019, it operated 1306 in-patient beds across 3 sites and had more than 1.7 million patient visits including 64,907 inpatient admissions and 276,003 emergency and urgent care visits. Due to their complexity, patients with CCN are often admitted to acute medicine services at both organizations and may require rehabilitation prior to returning home. We therefore focus our project in the general medicine and rehabilitation services at both organizations.

### Intervention

The Digital Bridge will be an integration of the Care Connector and ePRO technology solutions. Multimedia Appendices 1 and 2 offer descriptions of feature sets and wireframes of both solutions. Both technologies have been designed and developed through user-centered co-design approaches and have undergone usability and feasibility testing and evaluations [34,39-48].

The Digital Bridge will support care transitions by (1) inviting primary care physicians (PCPs) to access Care Connector while the patient is in hospital, allowing for asynchronous communication via the messaging feature for proactive discharge planning; (2) facilitating the inclusion of interprofessional recommendations in the discharge module (e.g., diet and mobility) typically missing from traditional physician-generated discharge summaries; (3) fostering electronic generation of PODS for use in patient-centered discharge teaching; (4) providing patients electronic access to PODS postdischarge to facilitate use of information at home; (5) adopting a digital enabled goal-oriented process to engage patients and families in the discharge process; and (6) providing ongoing self-management support for patients using ePRO for the vulnerable period 6 months postdischarge.

**Figure 1:**
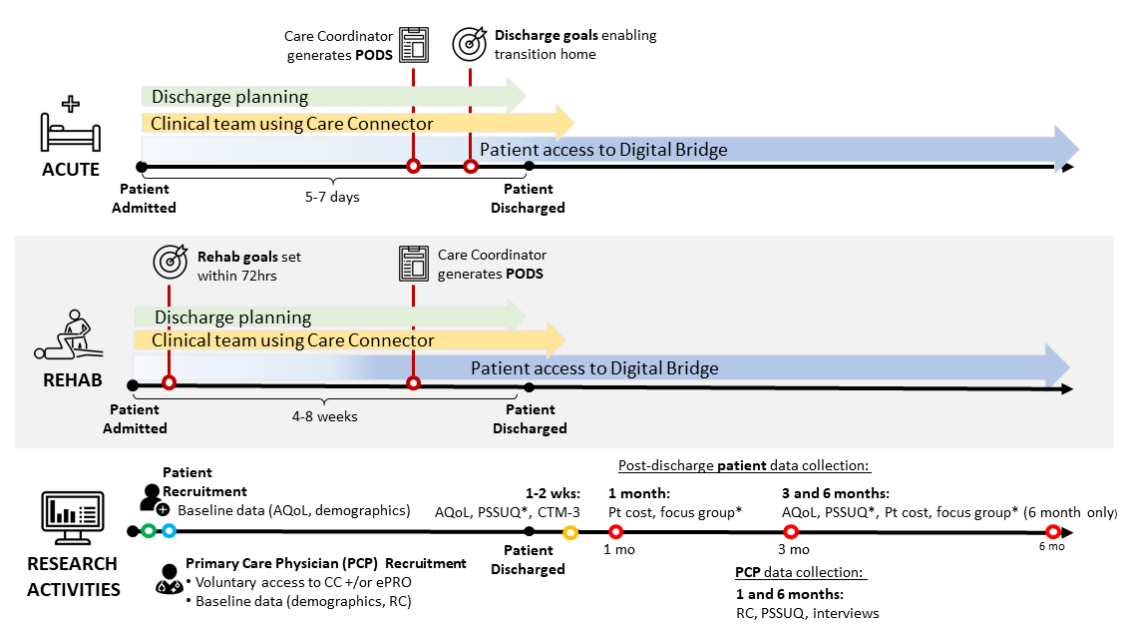
Proposed Digital Bridge workflow and trial data collection timelines.

While both systems have been tested and validated in their independent settings, we will co-design the workflow with end users to establish a feasible model for use in care transitions, modifying the technologies as needed. We will pay particular attention to integration with each hospital’s existing hospital information system (HIS) to support workflow and adoption. Of note, our hospital partners will use 3 distinct vendor HISs during the implementation phase. These HISs provide native support for the 6 functionalities to varying degrees. In our co-design, we will focus particularly on achieving the 6 functionalities above using the best technology (including those embedded in the local HIS) in each context to support workflow. We will use the term Care Connector to refer to technology functionalities 1 to 4, and ePRO to refer to technology functionalities 5 and 6. Although the transition workflow will be co-designed in phase 1, we anticipate the intervention will involve the process seen in Figure 1 and described in more detail in Multimedia Appendix 3.

## Methods

### Multiphased Evaluation Approach

The study includes two phases: workflow co-design (phase 1), followed by implementation and evaluation (phase 2). Research question 1 will be the main focus of phase 1, while phase 2 will address research questions 2 and 3. Table 1 offers an overview of the data collection strategy aligned to research questions and relevant theories and constructs.

**Table 1:** Data collection tools and timeline.

Research question, participant/level of analysis, and theories and constructs					Tool/method		Collection timeline
**Phase 1**							
	**What are the workflow design considerations in adopting digital solutions that bridge care settings to support transitions from hospital to home for patients with CCN^a^ from patient/caregiver, clinician, and organizational perspectives?**						
		**Patients, caregivers, and hospital and primary care providers**					
			User-centered co-design, usability and feasibility testing, FITT^b^ framework	4 working groups (2 hours each), cognitive walk-throughs, and PSSUQ^c^		12 weeks in year 1: 1 month for first 3 groups, 1 month for refining workflow, 1 month for final group, 1 group session including 5 to 10 walk-throughs, 2 to 3 weeks to finalize workflow (total 1 month)	
**Phase 2**							
	**Does the digital solution achieve quadruple aim goals by offering a cost-effective means of supporting care transitions to achieve improved provider experience (improved communication around transitions and improved teaming), patient experience with transitions (improved person-centered care transitions), and patient-reported outcomes (health-related quality of life)?**						
		**Patient (outcomes)**					
			Transition quality	CTM3^d^		1 to 2 weeks post-discharge	
			Health-related quality of life	AQoL-4D^e^		At 1 and 6 months post-discharge	
			Goal attainment	GAS^f^		As captured by ePRO^g^ (intervention only)	
		**Provider (processes): hospital and primary care**					
			Relational coordination	Relational coordination measure		Baseline, 1 and 6 months post-deployment (hospital providers) or first patient onboarded (primary care)	
			Quality of discharge summaries	Document analysis of PODS^h^ in Care Connector		Random sample via chart review	
		**Value for money**					
			Health system utilization and costs	ICES^i^		Utilization 1 year after discharge	
			Patient-reported costs	Patient cost survey		At 1 and 6 months post-discharge	
	**What are the implementation enablers and barriers to adopting technology in this process from patient, caregiver, provider, organizational, and system perspectives?**						
		**Patients and caregivers**					
			CFIR^j^ characteristics of individuals: demographics, level of complexity, social supports, comfort with technology	Patient information sheet		At recruitment	
			Self-efficacy, other relevant characteristics (eg, health literacy)	Focus groups (patients)		1 and 6 months post-discharge	
			CFIR process: patient-provider relationship, service frequency (what services from whom at what time points?)				
				Observation (discourse analysis)		Interactions in hospital and in community	
				Focus groups (patients)		1 and 6 months post-discharge	
		**Providers**					
			CFIR characteristics of individuals: demographics, profession, location, comfort with technology	Provider information sheet		At recruitment	
			CFIR process: provider workflows, provider-team communication	Observation		Training, onboarding, site visits	
		**All users (during implementation): CFIR characteristics of the intervention**					
			Usability of the tool	PSSUQ (survey)		1 and 6 months post-discharge	
			Data use	Digital Bridge system data use		Monthly	
			Perceived value and tool experience	Interviews (providers) and focus groups (patients)		1 and 6 months post-discharge	
			User interactions with tool	Observation and interviews		Training, patient and provider onboarding on technologies, and use during the study	
		**Organization(s)**					
			CFIR inner setting: hospital units, size, structure, resources, support, training, leadership, culture, and readiness to adopt				
				Document analysis		—	
				Interviews (providers)		Post-intervention	
			CFIR inner setting: primary care practices, size, structure, resources, support, training, leadership, culture, and readiness to adopt				
				Document analysis		—	
				Interviews (providers)		Post-intervention	
			CFIR process: change management	Interviews (providers)		1 and 6 months post-discharge	
		**System**					
			CFIR outer setting: system structure, standardization of data systems, legal requirements, funding, local resources, preexisting interorganizational linkages (particularly to primary care)	Interviews (providers)		Post-intervention	

^a^CCN: complex care needs.

^b^FITT: Fit between Individuals, Task, and Technology.

^c^PSSUQ: Post-Study System Usability Questionnaire.

^d^CTM-3: Care Transitions Measure–3.

^e^AQoL-4D: Assessment of Quality of Life–4 Dimensions.

^f^GAS: Goal Attainment Scale.

^g^ePRO: electronic Patient-Reported Outcome.

^h^PODS: Patient-Oriented Discharge Summary.

^i^ICES: Institute for Clinical Evaluative Sciences.

^j^CFIR: Consolidated Framework for Implementation Research.

#### Phase 1: Engaging in Co-Design Research to Adapt Validated Technologies Into New Contexts

Building on our previously successful co-design methods [34], we will establish patient/family, hospital provider, and primary care provider working groups (6-10 participants in each) to design the digitally enabled transition workflow. Starting with the intervention steps outlined above, each working group will work with the research team to modify the workflow. The research team will integrate modifications across the 3 groups and present this to a final working group consisting of a mix of representatives from the first 3 to finalize the workflow from all stakeholder perspectives. Each session will last approximately 2 to 3 hours. Care Connector and ePRO technologies will then be adapted and integrated into the Digital Bridge.

The co-design approach is consistent with other approaches that have sought to design new workflows that can be enhanced using existing technologies [49,50]. While this may reduce the ability to address all ideas that are arrived at through the co-design process, this approach does improve feasibility of the study as we do not have to build new technologies from scratch. We view this stage as part of the ongoing approach to co-design used by each technology in their respective design and building phases. As such, this project marks another important iterative step in the evolution of ePRO and Care Connector.

**Figure 2:**
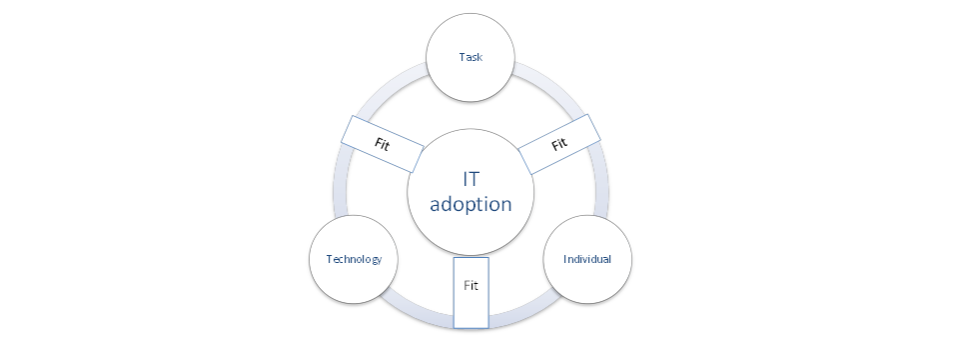
Fit between Individual Task and Technology framework (adapted from Ammenwerth [56]).

A feasibility and usability assessment will be conducted with working group members. Consistent with previous studies [39], we will adopt the Fit between Individuals, Task, and Technology (FITT) framework [51] to guide the data collection on feasibility (the ability for a technology to be adopted into a setting [52]) and usability (how well a technology meets user needs) [53-55] (see Figure 2 [56] for a visual of the FITT framework). Working group members will convene a final time and work in triads (hospital physician, PCP, and patient/family) to engage in a cognitive walk-through [57] of the Digital Bridge workflow. Triads will complete the 19-item Post-Study System Usability Questionnaire (PSSUQ) used to assess similar mobile health technologies [53] with demonstrated reliability and validity [58].

#### Phase 2: Implementation, Economic, and Developmental Evaluation

The evaluation is a pragmatic, real-world implementation and developmental evaluation design to support feasibility. A developmental evaluation approach, in which evaluation questions are used to support decision making and modifications to improve interventions and programs [59], allows for iterative modification of the intervention based on collected data. This method has been successfully adopted to evaluate the ePRO tool [46].

### Study Design

We will conduct a nonrandomized controlled trial to understand the impact of the intervention. For each site (SHS and THP) and each service (acute medicine and rehab), half of the participating wards will be designated as control while the other half will be designated as intervention (4 wards per hospital for a total of 8 wards). Quasi-experimental (or nonrandomized) design is often used in medical informatics evaluation due to insufficient sample size for cluster-randomized design [60] and complexity of intervention [61]. We have chosen to designate intervention wards together with operational leaders at each organization rather than randomize wards to increase the chance of successful implementation. While this could introduce selection bias into the study, it is felt this risk is worthwhile given that one important barrier to adoption of information communication technologies in hospitals is lack of readiness for implementation [62-64]. If clinical units that are not ready to take on a new technology implementation are randomized into the intervention group, we may experience a feasibility barrier that could derail the study before it begins. Our mixed-methods approach has been designed to capture important characteristics of the included clinical wards so that any potential selection bias can be understood.

General medicine units at both organizations serve a complex and clinically diverse population, with a median age of 73 (IQR 57-84) years and a median of 6 (IQR 3-9) coexisting medical conditions [65]. They care for patients with a wide variety of diagnoses (more than 200 distinct diagnoses) with the top 10 diagnoses comprising 36.2% of hospitalizations [66]. Rehabilitation services across the two organizations admit patients due to stroke, musculoskeletal concerns, and complex medical needs. At THP, services are not diagnosis-based; at SHS, however, patients are clustered on wards with specialized services.

We will collect baseline data from all wards (control and intervention) during phase 1 while co-design is ongoing and the intervention has not been deployed. During phase 2, after co-design is complete, the co-designed technology intervention and workflow will be rolled out to only the intervention wards. We will then collect data (identical to what was collected at baseline) from all wards (control and intervention) to understand the impact of technology by examining the differential change in control and intervention wards between baseline and intervention periods (difference in differences approach).

### Population and Recruitment

Patients will be recruited at the time of admission to one of the services (acute medicine or rehab) in the study. Patients aged 60 years and over, with CCN defined as presenting with 3 or more chronic conditions from the 16 most prominent in the population: arthritis (except rheumatoid arthritis), chronic coronary syndrome, dementia, hypertension, cardiac arrhythmia, rheumatoid arthritis, asthma, osteoporosis, stroke, depression, chronic obstructive pulmonary disease, acute myocardial infarction, diabetes, congestive heart failure, cancer, and renal failure.

This method is aligned with current and established methods to identify patients with CCN [67-69]. Patients must be slated to be discharged home. As the technology is only currently available in English, patients (or a caregiver) must be able to speak and read English. Patients with mild cognitive impairment will not be excluded if able to provide informed consent and engage with the intervention (independently or with caregiver aid). During phase 1, all recruited patients will receive usual care. During phase 2, recruited patients from the intervention wards will receive the Digital Bridge intervention while those from control wards will receive usual care. Family/caregivers of recruited patients may be invited to participate in qualitative interviews.

As workflow integration is a foundation for implementing technology, all hospital providers on the general medicine and rehabilitation services will be invited to participate. PCPs of recruited patients will be contacted and invited to participate while the patient is still in hospital. Recruited patients may still participate in the study if their PCP does not wish to participate or if they do not have a PCP at the time of study enrollment. Should the PCP choose to join, they will be consented and trained to adopt the technology to support their patients post-discharge if their patients were recruited from the intervention wards during phase 2.

### Study Measures

#### Developmental and Economic Evaluation Measures

Our primary outcome is the Care Transitions Measure–3 (CTM-3), a patient-reported measure of transition quality focusing on person-centeredness and communication. The CTM-3 has been validated in similar patient populations transitioning from hospital to home and primary care and in a systematic review of transitions measures was deemed to be the most acceptable measure of quality transitions [70]. To capture likely downstream impact, we will collect secondary outcomes including number of days at home (as a measure of readmissions), goals achieved (captured through the ePRO Goal Attainment Scale), and health-related quality of life (Assessment of Quality of Life–4 Dimensions [AQoL-4D], a brief survey validated in similar populations with demonstrated responsiveness and predictive validity with regard to entrance to long-term care [71,72]).

Team and provider level processes most relevant to this study are measures of communication and relational aspects of teamwork. Relational coordination is “a mutually reinforcing process of communicating and relating for the purpose of task integration” and is measured with a validated instrument consisting of 4 communication domains (frequency, timeliness, accuracy, problem solving) and 3 relational domains (shared goals, shared knowledge, respect) [73]. High relational coordination has been associated with improved patient functioning and quality of care and reduced pain and length of stay [74,75]. Relational coordination will be measured across 4 groups (hospital physicians, nursing, all allied health involved, and PCP) with care transition as the work process. We will administer the relational coordination survey to hospital clinicians at baseline and 1 and 6 months postdeployment. PCPs will be surveyed at enrollment and 1 and 6 months. We recognize using the Digital Bridge for care transitions may be rare events for individual PCPs; their views on communication and relationship with inpatient clinicians will be explicitly captured during interviews in addition to relational coordination surveys.

To determine cost-effectiveness in relation to outcome and process measures, an economic evaluation will be conducted to compare costs and outcomes among patients who transitioned out of hospital using Digital Bridge with the control patient group. Control and intervention patients will be followed for 1 year. Costs will be estimated from a societal perspective. Health services utilization and health system costs will be measured for 1 year using province-wide health system administrative data. Over 85% of total direct costs can be measured using a cost methodology for health administrative data implemented at ICES [76]. System utilization measures will include hospital admission, emergency department visits, days in acute care, 30-day readmissions, primary and specialty care visits, labs, diagnostic imaging, and 7-day post-discharge primary care follow-up. Out-of-pocket costs will be estimated using a patient survey. Caregiver time costs will be estimated using the average industrial wage. Total costs will include the sum of health system costs and out-of-pocket costs. Resources and costs required to design and implement Digital Bridge will be estimated through a project budget review.

#### Sample Size Calculation

Patient participants will be grouped into 1 of 4 arms (as determined by the unit they were discharged from) in both the baseline (pre-intervention) and postintervention periods. Our primary outcome, CTM-3, is scored as a continuous variable (0 to 100%) based on a published algorithm [77]. THP pilot data with 107 patients showed a baseline CTM-3 score of 74% (SD 21.4; unpublished). Our sample size calculation is based on an anticipated 13% to 14% increase in CTM-3 score and a standard deviation of 21.4 (derived from our previous studies of Care Connector). To ensure analysis for each of the 8 subgroups to be significant, we required a higher sample size than would be required in typical drug trials where randomization occurs at the individual level. As such we will recruit 33 to 38 patients per group; 8 groups in baseline (264 to 304) and 8 groups in intervention (264 to 304) for a total of 528 to 608 (based on *P*=.05 and power of .80).

#### Intervention Fidelity, Controlling for Bias, and Optimizing Internal Validity

We aim to optimize internal validity by asking providers about their involvement in other care transition interventions during the evaluation and in the 12 months prior to the study. We will also track readmitted patients who already used the Digital Bridge, as patients with CCN can have frequent hospital visits, without altering data collection time frame from index admission.

Our previous work identifies a need to balance real-world implementation and maintaining methodological rigor [78,79]. To accomplish this, we will distinguish core components of the intervention (what is determined to be central to success) from adaptable components [79]. This is an established approach to intervention fidelity in highly complex interventions such as this one [80,81]. Complex interventions often need to be continually adapted to local contexts and changing environments [82]. Attending to our third research question will support this work.

#### Implementation Measures

**Figure 3:**
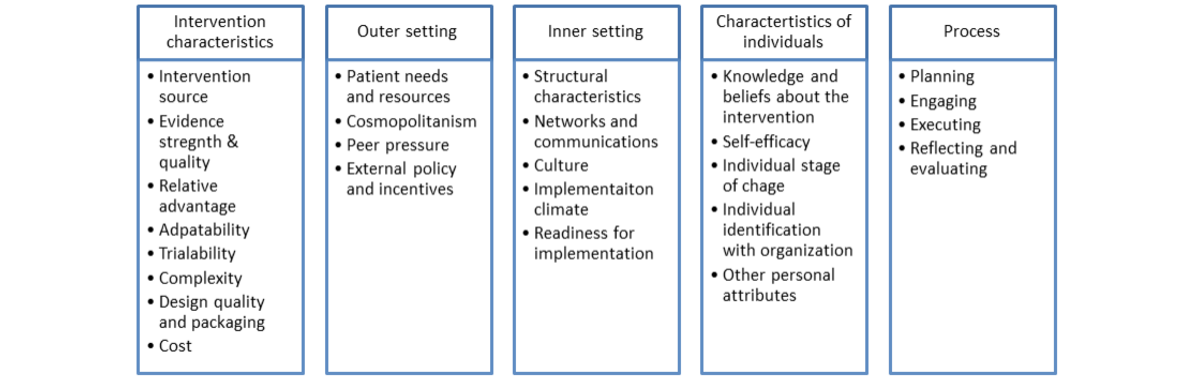
Consolidated framework for implementation research constructs (adapted from Damschroder [79]).

Implementation relates to the processes required to put an intervention or new model of care into use [79]. An implementation lens identifies context and process variables likely to influence intervention outcomes, known to be important in studies of digital health interventions [83]. Context and process data will additionally support the development of a scale and spread strategy [84] and is consistent with a developmental evaluation approach [59]. We will adopt the Consolidated Framework for Implementation Research (CFIR) [79], which compiles constructs that have been associated with effective implementation of complex interventions (see Figure 3 [79]).

An embedded ethnographic comparative case study methodology will be adopted, aligned with the case study approach of Yin [85] and the ethnographic approach to evaluating technology of Greehalgh [86]. Each site/service combination is considered a single case (8 cases total). Interviews with patients and caregivers (subset of 5 patients and caregivers from each unit, 40 patients and 40 caregivers total) and focus groups with providers and managers (6 to 8 participants per group [87]) will be conducted, along with a review of relevant organizational documents (eg, annual reports and vision statements) and observations of provider interactions and patient-provider interactions (eg, rounds, care planning meetings, clinic visits).

### Data Analysis Strategy

#### Phase 1: Workflow Analysis

Consistent with established user-centered co-design methods, we will adopt an interpretive descriptive approach to analyze focus group data and iteratively design the workflow [88]. Analysis will guide modifications and integration of the Care Connector and ePRO technologies into the Digital Bridge. Feasibility and usability will be assessed using standard descriptive statistics across 3 domains of the PSSUQ. Demographic information collected from participants will inform analysis and support transferability to other settings.

#### Phase 2: Developmental Evaluation and Implementation Analysis

**Figure 4:**
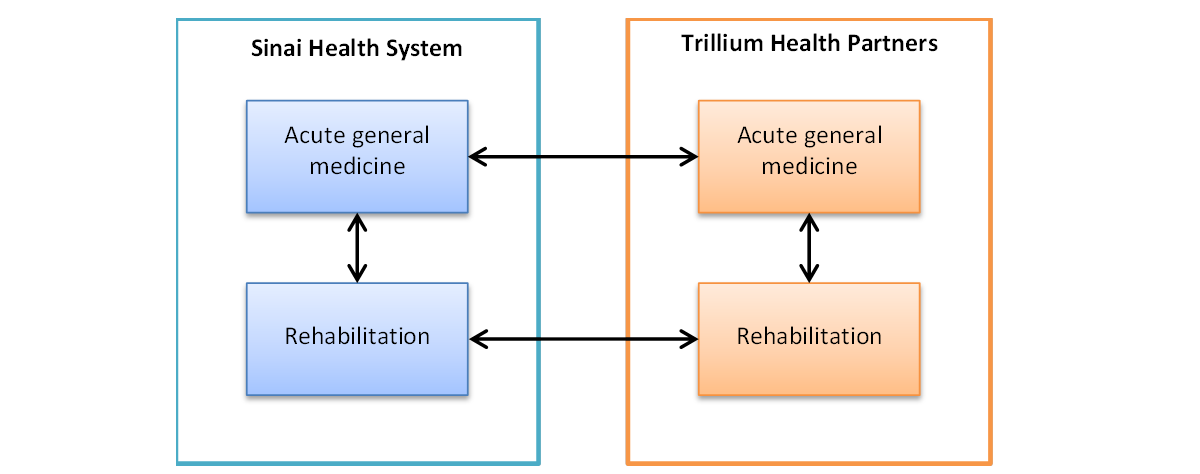
Within and between group pre-post analysis.

We will adopt a multimethod case study analysis approach [85], looking at within and between group assessments (Figure 4), accounting for hospital/service specific context variables. Single case and cross-case comparative analyses will be used to assess context, process, and outcome measures.

For quantitative outcome measures (eg, CTM-3, relational coordination, AQoL-4D), statistical comparisons between the pre- (baseline) and post- (intervention) data will be made using mixed-effects regression models to account for potential clustering effect of patients from the same service. As this is a complex intervention and local context/implementation factors may affect outcomes, we will perform sensitivity analysis and examine each of the participating services separately. For repeated measures within the same individual, we will use a difference in differences approach (eg, AQoL-4D measured at 0, 2 weeks, 1 month, and 6 months post-discharge). Patients may experience decline in QoL over time in both control and intervention groups for reasons unrelated to the intervention; a lesser magnitude of decline in intervention versus control group may suggest a benefit of the intervention.

For patients who do not complete a study follow-up, we will analyze all available data points. Additionally, understanding the characteristics (via collected demographic and administrative data of this study) of those who do not complete follow-up may inform us of under what conditions and for whom the technology works best.

Qualitative data will be analyzed using an ethnographic case study approach [86], adopting techniques such as interpretive descriptive coding methods and word tables [85] to support the within and cross case analysis as used in previous evaluations and similar case comparisons [33,48]. Two researchers trained in qualitative research will read interview and focus group transcripts and field notes, record key themes, compare, and discuss findings. Both inductive and deductive analysis techniques will be used to identify themes and then conceptually map with the CFIR theoretical framework. NVivo software (QSR International) will be used for qualitative data management.

#### Phase 2: Economic Evaluation Analysis

A cost utility analysis will be conducted comparing total costs and quality-adjusted life years (QALYs) of Digital Bridge to usual care from the perspective of Canada’s health care system. Costs and utility values will be assessed over the study follow-up period. Analysis will conform to most recent Canadian guidelines for economic evaluation [89]. Responses to the AQoL-4D will be scored using preference weights, converting the 5 responses into a single summary index, where a score of 1 reflects perfect health and 0 is equivalent to dead [90]. QALYs will be estimated using total area under the curve methods [91]. Statistical analysis will be conducted in accordance with current guidelines for clinical and cost-effectiveness analysis alongside randomized controlled trials [92], accounting for the repeated nature of the cost and outcome data. The incremental cost and QALYs will be estimated using generalized estimating equations, a flexible multivariate regression framework that explicitly allows for the modeling of nonnormal distributional forms of repeated measure data. As we will have individual level data on costs and QALYs for the period of study follow-up, we will evaluate uncertainty of the cost utility estimates using nonparametric bootstrapping, obtaining 5000 estimates of costs and QALYs for each strategy. Bootstrapping results will also be used to estimate 95% confidence intervals and depict cost-effectiveness acceptability curves. These analyses represent the probability of an intervention being cost-effective to a range of potential threshold values that the health system may be willing to pay for an additional unit of effect. As a scenario analysis, we will also conduct a cost utility analysis from a societal perspective by including patient-related costs. Economic analysis will be conducted using Stata version 15.1 (StataCorp LLC) and Excel Visual Basic for Applications (Microsoft Corporation). Analyses will support a proposed model of scale and spread in the Ontario context and/or similar health system contexts in Canada based on patient volumes.

### Ethical Considerations and Dissemination

The study will undergo research ethics board review and receive approval at each study site. All participants will provide signed informed consent prior to participation. The study was registered at ClinicalTrials.gov [NCT04287192].

An integrated knowledge translation strategy will be implemented; engaging stakeholders and knowledge users in the design, implementation and interpretation of study results. A Knowledge Translation Advisory Group will be established to provide insight and feedback regarding the project design and implementation, leading the development of the project knowledge translation strategy and associated outputs. The advisory group will comprise our project team, collaborators, patient representatives, and decision-making partners. Knowledge translation activities will ensure that the results are made available to those who need them and are packaged in a manner for sustained knowledge use; presentations at relevant conferences and in peer-reviewed publications and a symposium at project end are planned. A key knowledge translation activity is the development of a scale and spread strategy which will identify spread sites for the intervention in other hospitals in Ontario and other provinces across Canada.

## Results

The project began August 2019 and received ethics approval from all necessary institutions September 2020. Due to challenges related to the COVID-19 pandemic, we have adjusted our timelines and anticipate the project will be completed by Spring 2024. This study was funded by the Canadian Institute for Health Research through a Team Grant in Transitions in Care (FRN 165733). Results from different phases will be published in peer-review journals. Phase 1 results are expected to be published in 2022, with phase 2 findings published in 2025.

## Discussion

### Summary

This protocol describes a novel approach to developing, implementing, and evaluating a digital health technology to support transitions of complex patients returning home from hospital. By incorporating a mixed-methods and pragmatic approach we will be able to purposefully develop, feasibly implement, and rigorously evaluate the Digital Bridge solution. The study seeks to determine if the Digital Bridge will support improved communication across providers in the hospital and community and the patient and family caregivers, leading to greater shared situational awareness during care transitions.

### Strengths and Limitations

Given the real-world implementation of Digital Bridge, practice changes at the research sites and variable adherence to the implementation protocols are likely. Additionally, contamination between control and intervention wards may occur as clinicians rotate between units and modify their behaviors in control settings based on using Digital Bridge in intervention settings. Capturing and understanding these considerations is essential in order to identity the range of factors that may have influenced study results. In addition, the health system within which the study is being conducted is undergoing transformational change, and the research team must track any policy and organizational changes that may affect the implementation.

Other important limitations to track and potentially mitigate with regard to the intervention focus on integration of the solution into practice-based electronic medical record (EMR) systems. While Care Connector is integrated with the hospital-based EMR, we may not be able to fully integrate the Digital Bridge (which includes the ePRO tool) into primary care EMRs. A lack of interoperability can lead to tools not aligning to clinical processes and additional burden on providers, a challenge that has been well documented in the implementation literature [93]. We will probe the impact on workflow as part of our interviews with providers using the tool to determine the degree of impact. We will also use implementation findings to assess generalizability of findings to other nonurban settings. Given the intervention will be running in large urban health care organizations, findings may not be fully transferable to other small nonurban settings.

One important limitation of this study is in the separation of groups (patients, families, clinicians) in the first round of co-design working groups. Based on previous experience co-designing with mixed groups, we have found that bringing groups together too early in the process can lead to conversations being dominated by those perceived as holding the most power and knowledge, often physicians. We decided to forgo the opportunity to create shared understanding early in the process in favor of creating safe spaces in which participants feel able to speak freely and openly about what works and what doesn’t for them. It is our hope that bringing the groups together after an initial engagement will help participants feel more comfortable with the process and able to speak freely in mixed groups where shared understanding can be built.

The protocol has a number of strengths. First, the use of a pragmatic trial will allow for adaptability required in real-world complex interventions, while the ethnography will uncover core and adaptable factors. Second, using co-design approaches will help adapt the model to local contexts and ensure that study results meet the needs of stakeholders. Third, broad inclusion criteria and minimal exclusion criteria will enable researchers to capture as much variation in the patient population as possible. And finally, the multisite nature of the study will support broader application of study results. A potential challenge will be ensuring we have adequate representation of the wider patient population in co-design activities. Working with patient and family representatives as well as the patient engagement offices at each research site will help mitigate this concern. With platforms currently only available in English, a language barrier to access may be created. The Knowledge Translation Advisory Committee (which will include patients and family representatives) will help determine the extent of that barrier and mitigate as best possible throughout the implementation.

### Potential Impact

With wider adoption of the proposed intervention, this project could have impact at patient, clinician, organizational, and health system levels. Patients and families may have improved experience with transitions and health-related quality of life. Clinicians may experience greater efficiency in their coordination of care efforts and likely fewer errors and missed information. For organizations, the Digital Bridge could help standardize care transition practices across organizational boundaries. Finally, at the health system level, the Digital Bridge could address the growing challenge of transitioning older adults with CCN from hospital to home, potentially reducing unnecessary readmission or emergency department visits by patients post-discharge, leading to cost savings.

Should the intervention evaluation come out favorably, future work will also seek to test the Digital Bridge’s ability to support transitions for patients to other settings such as long-term care, assistive living, or long-term rehabilitation.

## Appendix

Multimedia Appendix 1 Care Connector features and wireframes.

Multimedia Appendix 2 Electronic Patient-Reported Outcome features and wireframes.

Multimedia Appendix 3 Description of Digital Bridge workflow.

Multimedia Appendix 4 Peer Review Comments.

## Abbreviations

AQoL-4D: Assessment of Quality of Life–4 Dimensions
CCN: complex care needs
CFIR: consolidated framework for implementation research
CTM-3: Care Transitions Measure–3
EMR: electronic medical record
ePRO: electronic Patient-Reported Outcome
FITT: Fit between Individuals, Task, and Technology
HIS: hospital information system
ICES: Institute for Clinical Evaluative Sciences
PODS: Patient-Oriented Discharge Summaries
PCP: primary care physician
PSSUQ: Post-Study System Usability Questionnaire
QALY: quality-adjusted life year
SHS: Sinai Health System
THP: Trillium Health Partners
