# Supplementary material for: Building a Digital Bridge to Support Patient-Centered Care Transitions From Hospital to Home for Older Adults With Complex Care Needs: Protocol for a Co-Design, Implementation, and Evaluation Study
Source: JMIR Res Protoc. 2020 Nov 25;9(11):e20220. doi: 10.2196/20220 (PMC7725647; doi:10.2196/20220)

Multimedia Appendix 1: Care Connector features and wireframes

Care Connector is an electronic inter-professional communication and collaboration platform designed and developed at THP through continuous engagement with frontline clinicians in user co-design combined with agile software development[1]. Care Connector integrates with and complements the hospital health information system (HIS) by providing communication and collaboration features (e.g. inter-professional care planner, secured asynchronous messaging) that fit with clinicians' workflow. It has been in operation at THP since 2015. A mixed methods study evaluating its impact showed improvement in relational and communication aspects of teamwork in some settings [2]. A discharge module supporting care transitions has also been deployed, including a patient-facing component (e.g. PODS). While Care Connector addresses initial discharge planning and communication/collaboration needs in the care transition process, it does not provide ongoing support to patients after transition to the community when they are most vulnerable and more likely to experience unnecessary ED visits and readmissions. Integration of the ePRO tool into this workflow can address this gap, providing ongoing communication and self-management support after patients return home.

1. Tang, T., et al., *Clinician user involvement in the real world: Designing an electronic tool to improve interprofessional communication and collaboration in a hospital setting.* International Journal of Medical Informatics, 2018. **110**: p. 90-97.
2. Tang, T., et al., *Using an electronic tool to improve teamwork and interprofessional communication to meet the needs of complex hospitalized patients: A mixed methods study.* International journal of medical informatics, 2019. **127**: p. 35-42.

Care Connector Feature Set

Physician Sign-Out	A structured communication tool supporting physician handover during times of coverage or service change using the evidence based I-PASS methodology (Starmer et al, NEJM 2014)
Progress Note	Supports efficient workflow by enabling clinicians to produce clinical documentation while completing other tasks in the system (e.g. updating the Physician Sign-Out, or the Interprofessional Care Planner).
Interprofessional Care Planner	A succinct electronic summary of the patient's current medical, nursing, psychosocial, and functional information all on one page that is accessible by any member of the team and is updated by all disciplines. Nurses use this for handover twice per day and other disciplines use this summary to understand the patient's status and progress from other disciplines' perspectives.
Flow Planner	Supports flow team in tracking tasks and planning for discharge
Messaging	Teams use this feature to send non-time-sensitive patient related messages to reduce unnecessary interruptions. Messages are visible to the entire team and not restricted to sender/receiver.
Discharge Summary	Supports generation of a comprehensive structured discharge summary (design guided by feedback from local primary care physicians) and a patient-oriented discharge summary (PODS) (Hahn-Goldberg J Hosp Med 2015) with input by the interprofessional team.

Care Connector Wire-Frames

<u>Care Connector Provider Application - Screen Shots</u>

The following are screenshots from the application that is operational at Trillium Health Partners. Data shown are fabricated and do not represent real patient data.

<u>Log-in Screen</u>

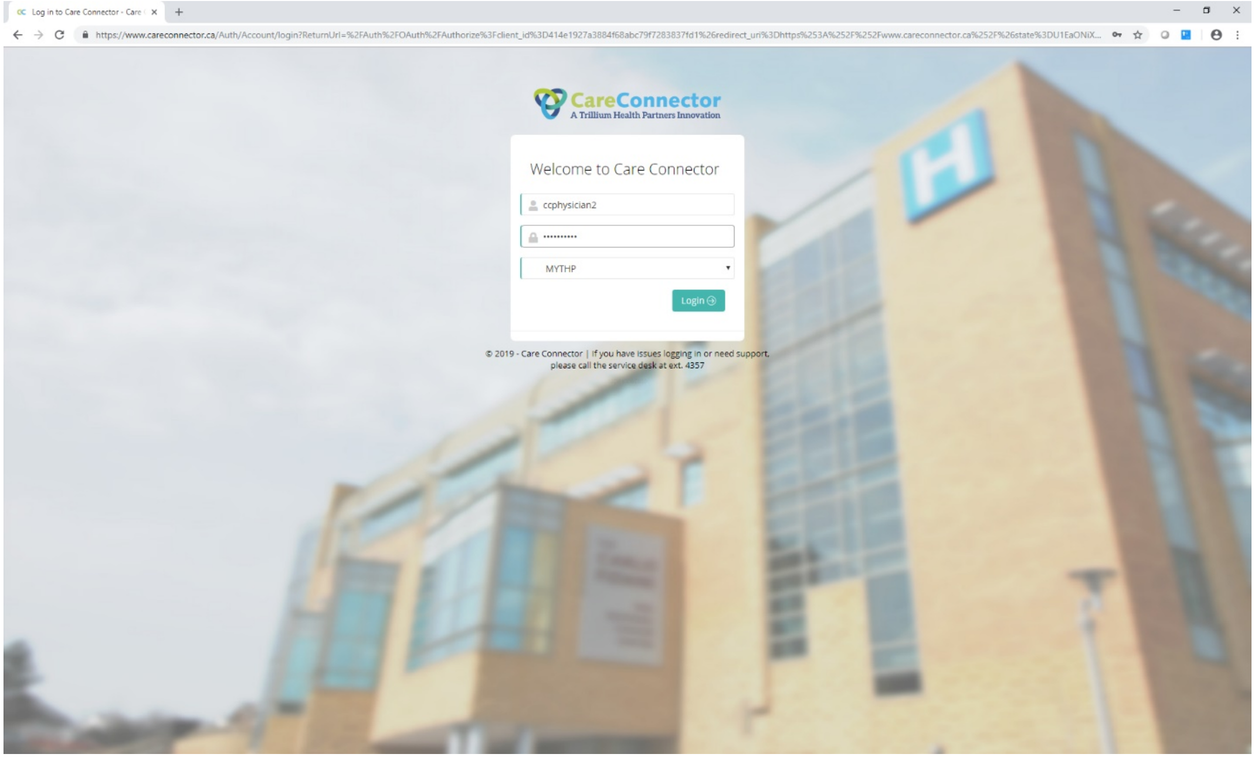

Patient Details

This screen allows healthcare providers to complete the official discharge summary and the information that will be included in the Patient Oriented Discharge Summary (PODS).

Note other functionalities (see Messaging circled in red) that allow care teams to communicate and collaborate.

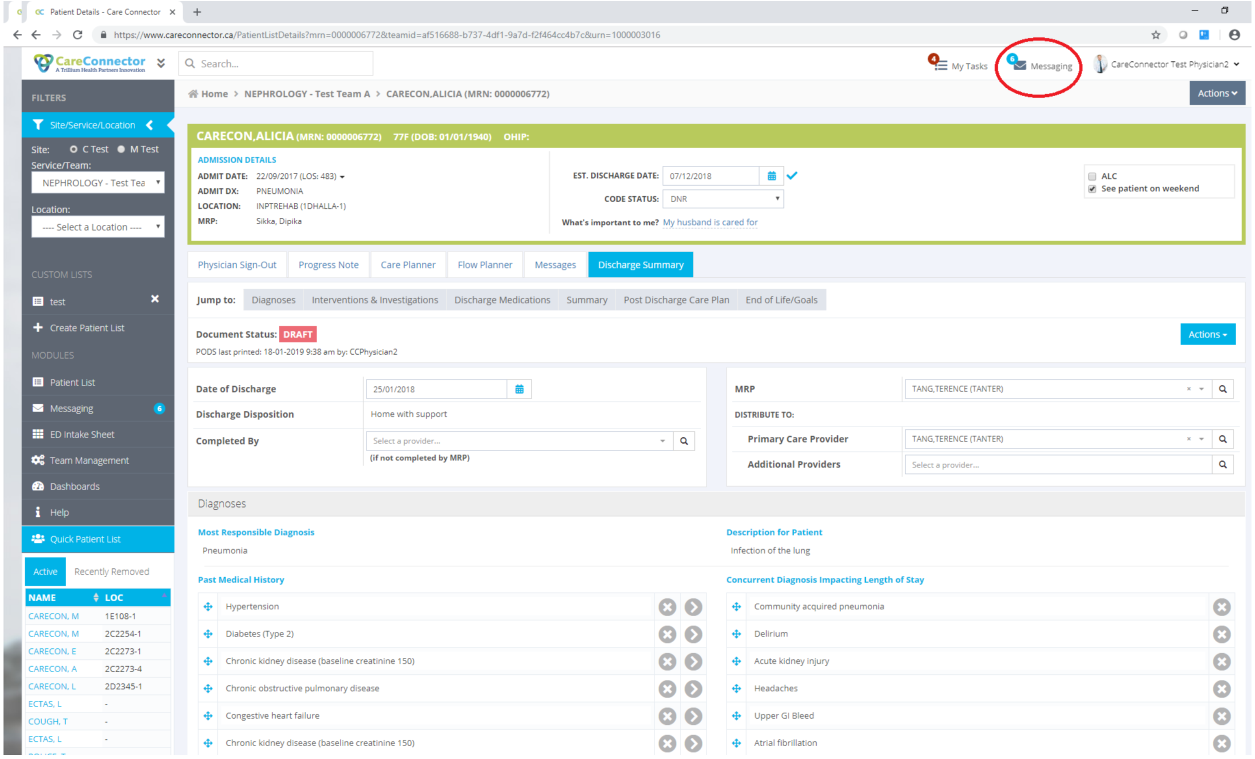

<u>Patient Oriented Discharge Summary</u>

Printing of the Patient Oriented Discharge Summary (PODS).

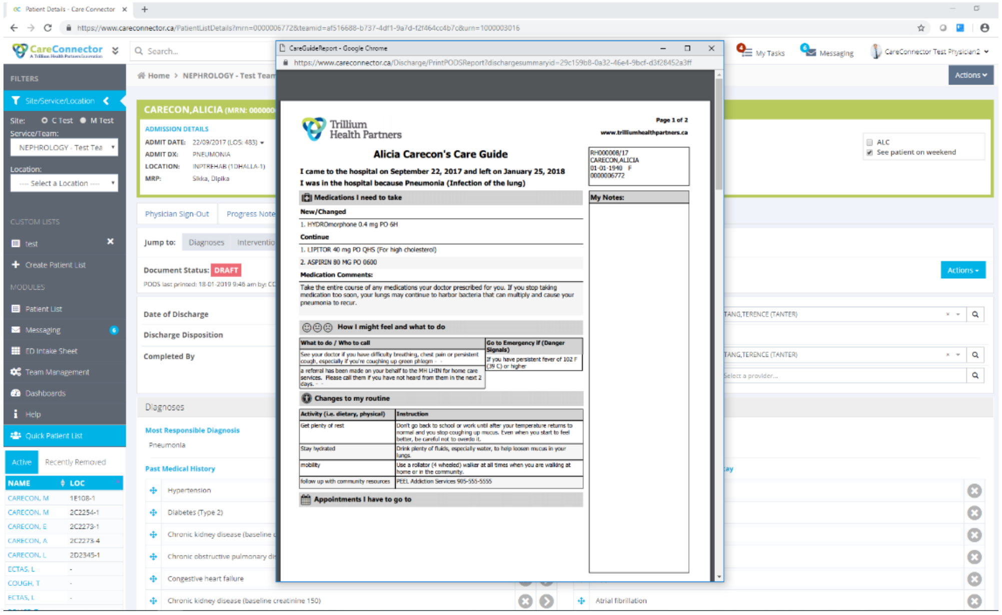

Example of a generated PODS (see next 2 pages)

Trillium Health Partners
www.trilliumhealthpartners.ca

Alicia Carecon's Care Guide

RH000008/17
CARECON,ALICIA
01-01-1940 F
0000006772

I came to the hospital on September 22, 2017 and left on January 25, 2018

I was in the hospital because Pneumonia (Infection of the lung)

Medications I need to take

New/Changed

1. HYDROmorphone 0.4 mg PO 6H

Continue

1. LIPITOR 40 mg PO QHS (For high cholesterol)
2. ASPIRIN 80 MG PO 0600

Medication Comments:

Take the entire course of any medications your doctor prescribed for you. If you stop taking medication too soon, your lungs may continue to harbor bacteria that can multiply and cause your pneumonia to recur.

How I might feel and what to do

What to do / Who to call	Go to Emergency if (Danger Signals)
See your doctor if you have difficulty breathing, chest pain or persistent cough, especially if you're coughing up green phlegm - -	If you have persistent fever of 102 F (39 C) or higher
a referral has been made on your behalf to the MH LHIN for home care services. Please call them if you have not heard from them in the next 2 days. - -	

Changes to my routine

Activity (i.e. dietary, physical)	Instruction
Get plenty of rest	Don't go back to school or work until after your temperature returns to normal and you stop coughing up mucus. Even when you start to feel better, be careful not to overdo it.
Stay hydrated	Drink plenty of fluids, especially water, to help loosen mucus in your lungs.
mobility	Use a rollator (4 wheeled) walker at all times when you are walking at home or in the community.
follow up with community resources	PEEL Addiction Services 905-555-5555

Appointments I have to go to

My Notes:

1102 D HR

RH000008/17
CARECON,ALICIA
01-01-1940 F
0000006772

Alicia Carecon's Care Guide

I came to the hospital on September 22, 2017 and left on January 25, 2018

I was in the hospital because Pneumonia (Infection of the lung)

Who to see	reason	date and time	Location	phone number	booked?
Nephrologist	Check your kidney	Thu Dec 20 2018 09:00:00 GMT-0500 (Eastern Standard Time)	123 Somewhere drive	(905) 555-5555	Y
Diabetes Education Centre		Thu Jan 24 2019 11:15:00 GMT-0500 (Eastern Standard Time)	2300 Eglinton Ave. West, Mississauga	905-813-1576	Y
Heart Function Clinic			Credit Valley Hospital	905-813-2712	
CT scan	to check your brain		Trillijum DI	905-843 0000	
Community Care Access Centre				905-310-CACC (2222)	

Where to go for more information

Action	Timeframe
Get vaccinated: Vaccines are available to prevent some types of pneumonia and the flu. Talk with your doctor about getting these shots. The vaccination guidelines have changed over time so make sure to review your vaccination status with your doctor even if you recall previously receiving a pneumonia vaccine.	

My Hospital care Team

Most Responsible Physician (MRP): TANG,TERENCE

1102 D HR

Care Connector Patient Application – Wireframe

The following are conceptual drawings of the patient application that is currently under development. Expected launch date is Spring 2019.

Welcome Screen

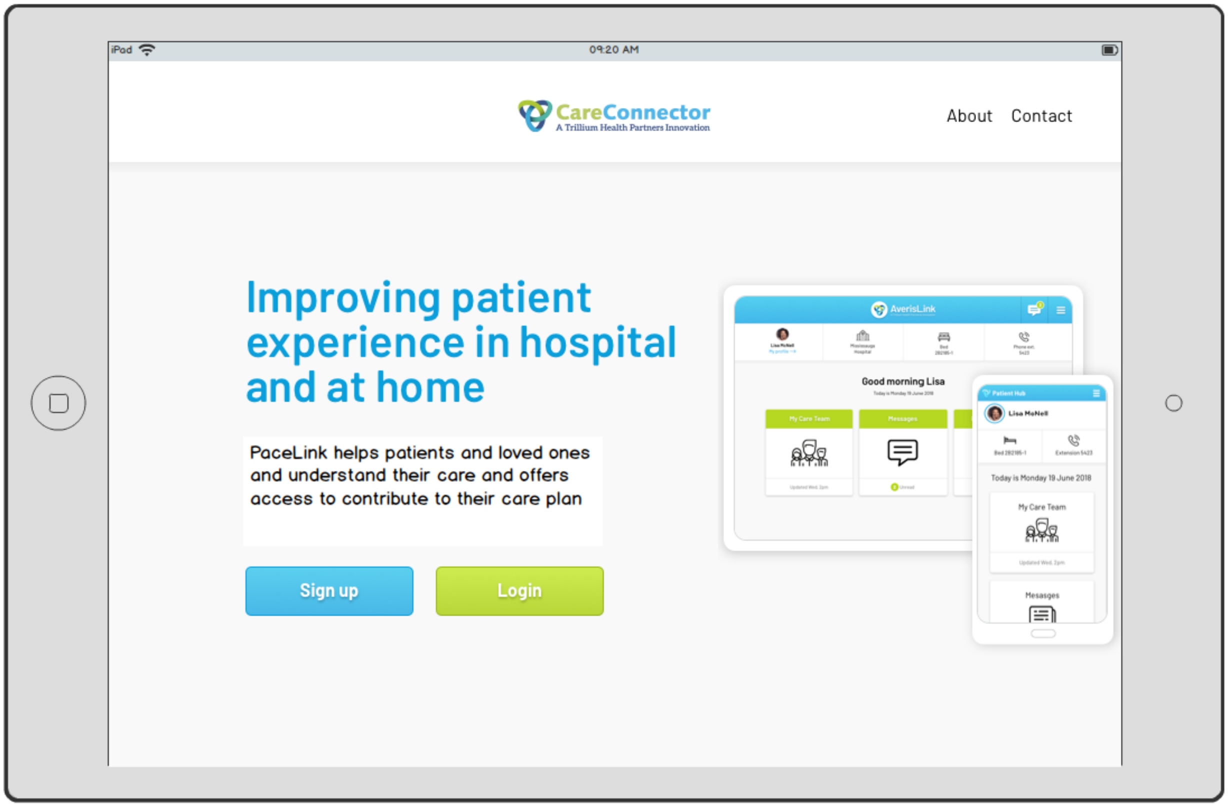

Main Screen

This allows user to view care plans (including patient oriented discharge summary [PODS]) that has been shared with them.

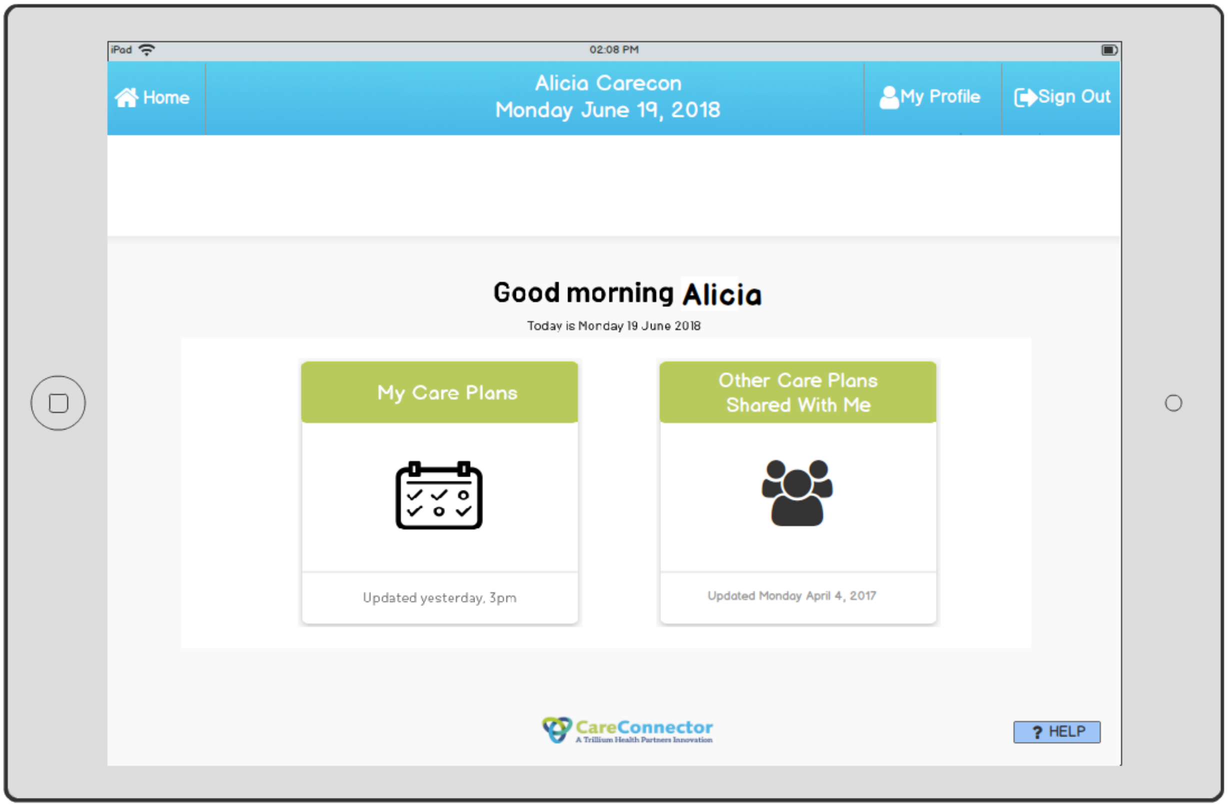

Patient View of the Discharge Care Plan

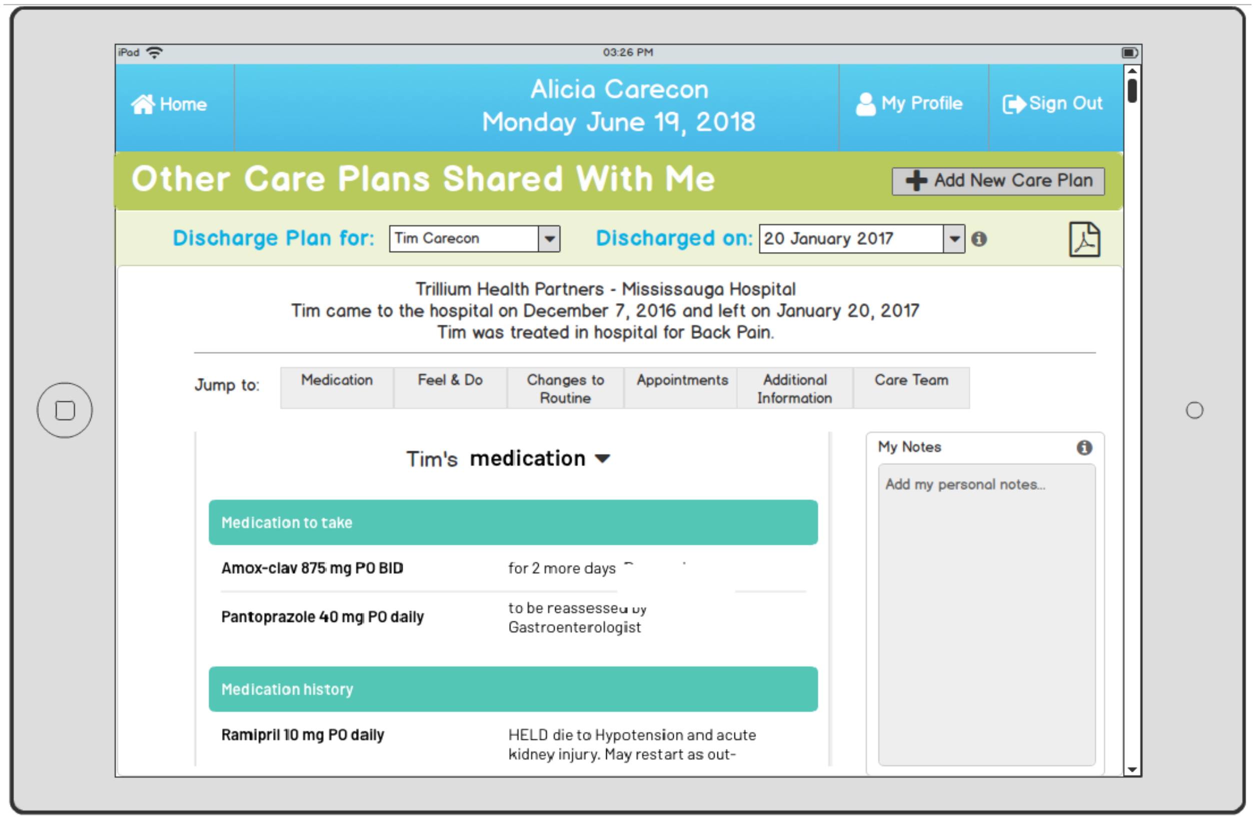

Supplement: Multimedia Appendix 1 [file resprot_v9i11e20220_app1.pdf]
