# Supplementary material for: Building a Digital Bridge to Support Patient-Centered Care Transitions From Hospital to Home for Older Adults With Complex Care Needs: Protocol for a Co-Design, Implementation, and Evaluation Study
Source: JMIR Res Protoc. 2020 Nov 25;9(11):e20220. doi: 10.2196/20220 (PMC7725647; doi:10.2196/20220)
Supplement: Multimedia Appendix 2 [file resprot_v9i11e20220_app2.pdf]

Multimedia Appendix 2: ePRO features and wireframes

The *ePRO Tool* has been iteratively co-designed, tested and evaluated with patients with CCN, their caregivers, and their primary care physicians (PCPs) in Ontario since 2013. User-centred co-design methods and iterative rapid real-world implementation has guided this work [1-4]. Full descriptions of the design and development and the evaluation protocol have been published [5-9]. The ePRO tool includes two features: 1) My Goal Tracker and 2) Health Journal. (visit https://www.eprobridgepoint.com/ to view ePRO in action). *My Goal Tracker* allows patients and providers to collaboratively create goal-oriented patient care-plans, and track outcomes related to their goals using a mobile device. SMART (Specified-Measureable-Attainable-Realistic-Time Specific) goal principles guide goal set-up, and Goal-Attainment Scaling is used to measure outcomes, and is argued to be the most appropriate standardized outcome measure for older adults with CCN [10] [11]. Customizable monitoring questions can be added, and patients can include comments to provide context needed to understand why goals are, or are not, achieved. *Health Journal* helps patients, their caregivers and PCP monitor symptoms and outcomes most relevant to this patient group [8]. Patients, caregivers and providers track goal progress and view patient data on the mobile device or portal.

1. Brown, A.L., *Design experiments: Theoretical and methodological challenges in creating complex interventions in classroom settings.* The Journal of the Learning Sciences, 1992. **2**(2): p. 141-178.
2. Collins, A., *Toward a Design Science of Education*, in *New directions in educational technology*, E.S.T. O'Shea, Editor. 1992, Springer-Verlag: Berlin.
3. Devi, K.R., A.M. Sen, and K. Hemachandran, *A working Framework for the User-Centered Design Approach and a Survey of the available Methods.* International Journal of Scientific and Research Publications, 2012. **2**(4): p. 1-8.
4. Collins, A., D. Joseph, and K. Bielaczyc, *Design Research: Theoretical and Methodological Issues.* Journal of the Learning Sciences, 2004. **13**(1): p. 15-42.
5. Steele Gray, C., et al., *Improving Patient Experience and Primary Care Quality for Patients With Complex Chronic Disease Using the Electronic Patient-Reported Outcomes Tool: Adopting Qualitative Methods Into a User-Centered Design Approach.* JMIR Res Protoc, 2016. **5**(1): p. e28.
6. Steele Gray, C., et al., *Supporting Goal-Oriented Primary Health Care for Seniors with Complex Care Needs Using Mobile Technology: Evaluation and Implementation of the Health System Performance Research Network, Bridgepoint Electronic Patient Reported Outcome Tool* JMIR Res Protoc, 2016. **5**(2): p. e126.
7. Steele Gray, C., et al., *The Electronic Patient Reported Outcome Tool: Testing Usability and Feasibility of a Mobile App and Portal to Support Care for Patients With Complex Chronic Disease and Disability in Primary Care Settings.* JMIR Mhealth Uhealth, 2016. **4**(2): p. e58.
8. Steele Gray, C., et al., *Tying eHealth Tools to Patient Needs: Exploring the Use of eHealth for Community-Dwelling Patients With Complex Chronic Disease and Disability.* JMIR Res Protoc, 2014. **3**(4): p. e67.
9. Cresswell, K. and A. Sheikh, *Organizational issues in the implementation and adoption of health information technology innovations: an interpretative review.* International journal of medical informatics, 2013. **82**(5): p. e73-e86.
10. Bovend'Erdt, J., R. Botell, and D. Wade, *Writing SMART rehabilitation goals and achieving goals attainment scaling: a practical guide. .* Clinical Rehabilitation, 2009. **23**: p. 352-361.
11. Reuben, D.B. and M.E. Tinetti, *Goal-Oriented Patient Care — An Alternative Health Outcomes Paradigm.* New England Journal of Medicine, 2012. **366**(9): p. 777-779.

ePRO Wire-Frames

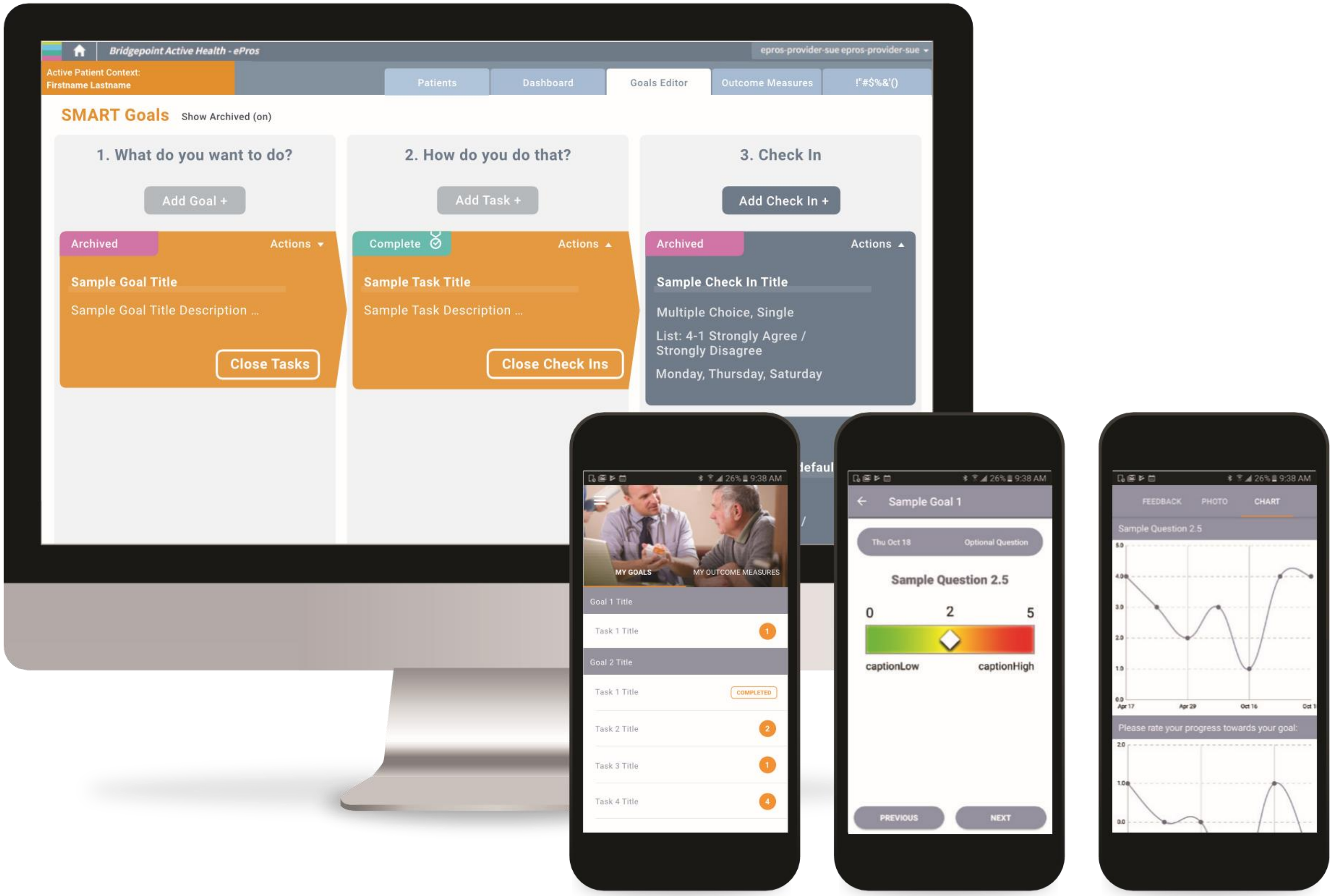

Patient registration on system

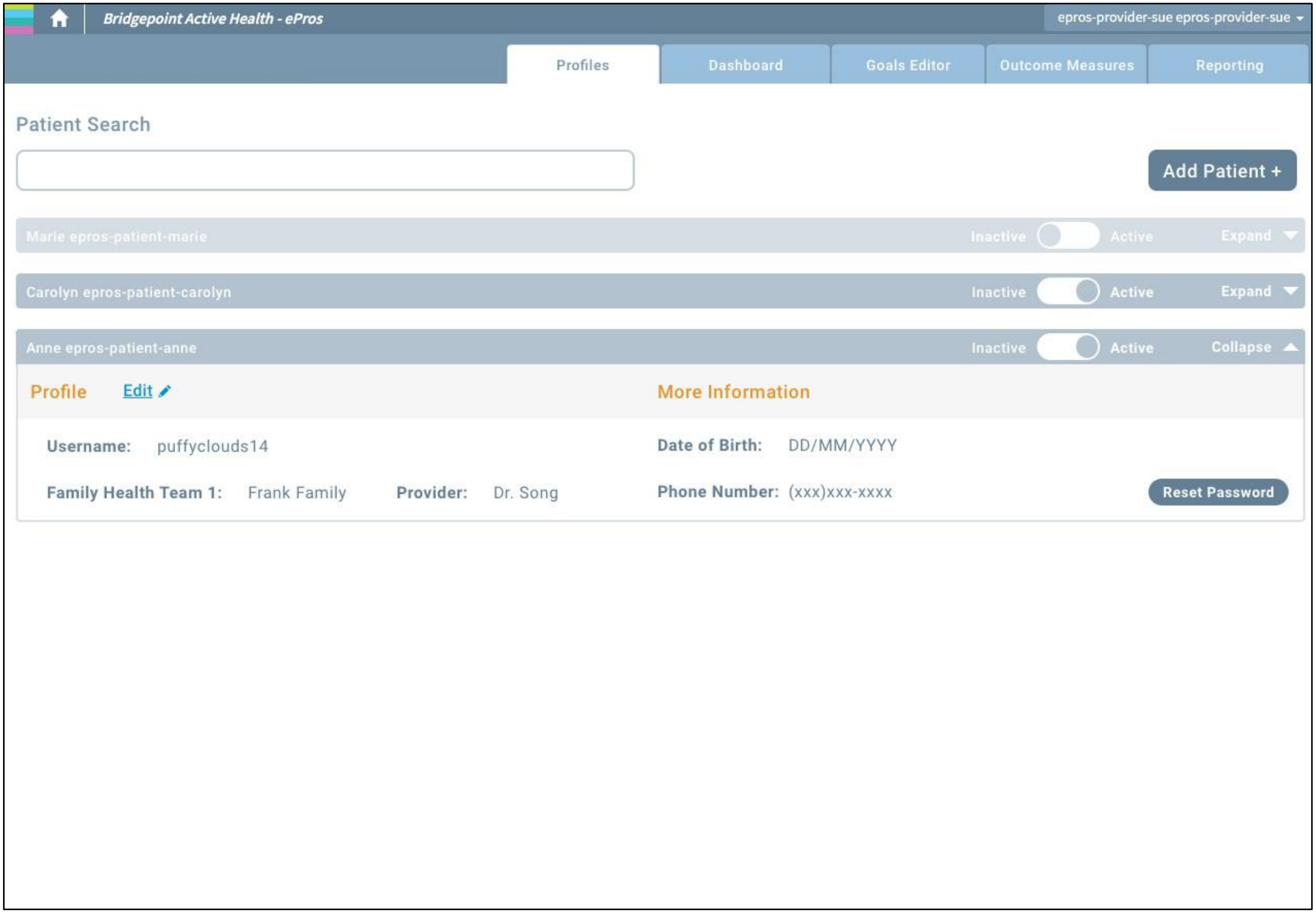

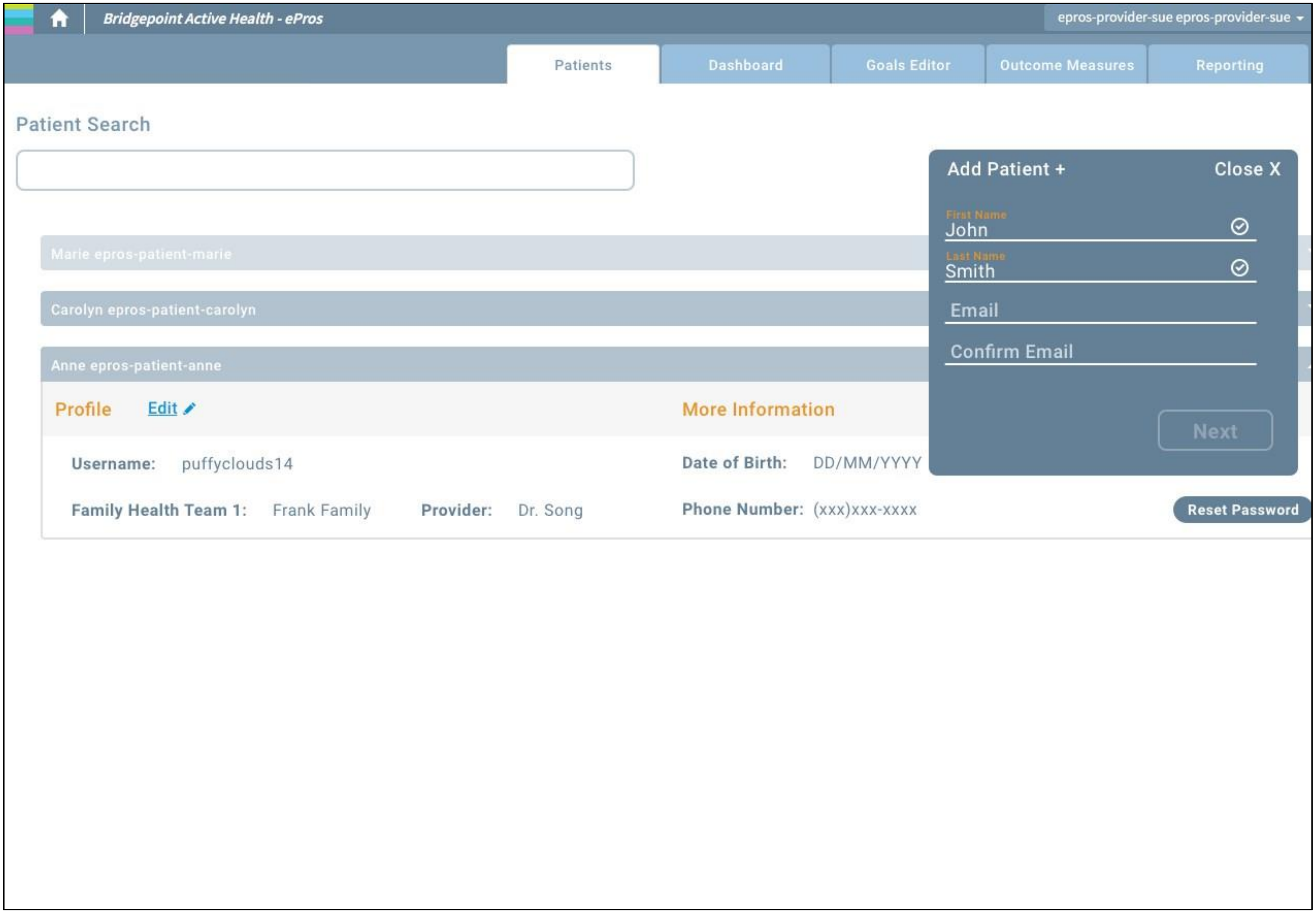
Bridgepoint Active Health - ePros
epros-provider-sue epros-provider-sue
Patients
Dashboard
Goals Editor
Outcome Measures
Reporting
Patient Search
Marie epros-patient-marie
Carolyn epros-patient-carolyn
Anne epros-patient-anne
Profile
Edit
More Information
Username: puffyclouds14
Date of Birth: DD/MM/YYYY
Family Health Team 1: Frank Family
Provider: Dr. Song
Phone Number: (xxx)xxx-xxxx
Reset Password
Add Patient +
Close X
First Name
John
Last Name
Smith
Email
Confirm Email
Next

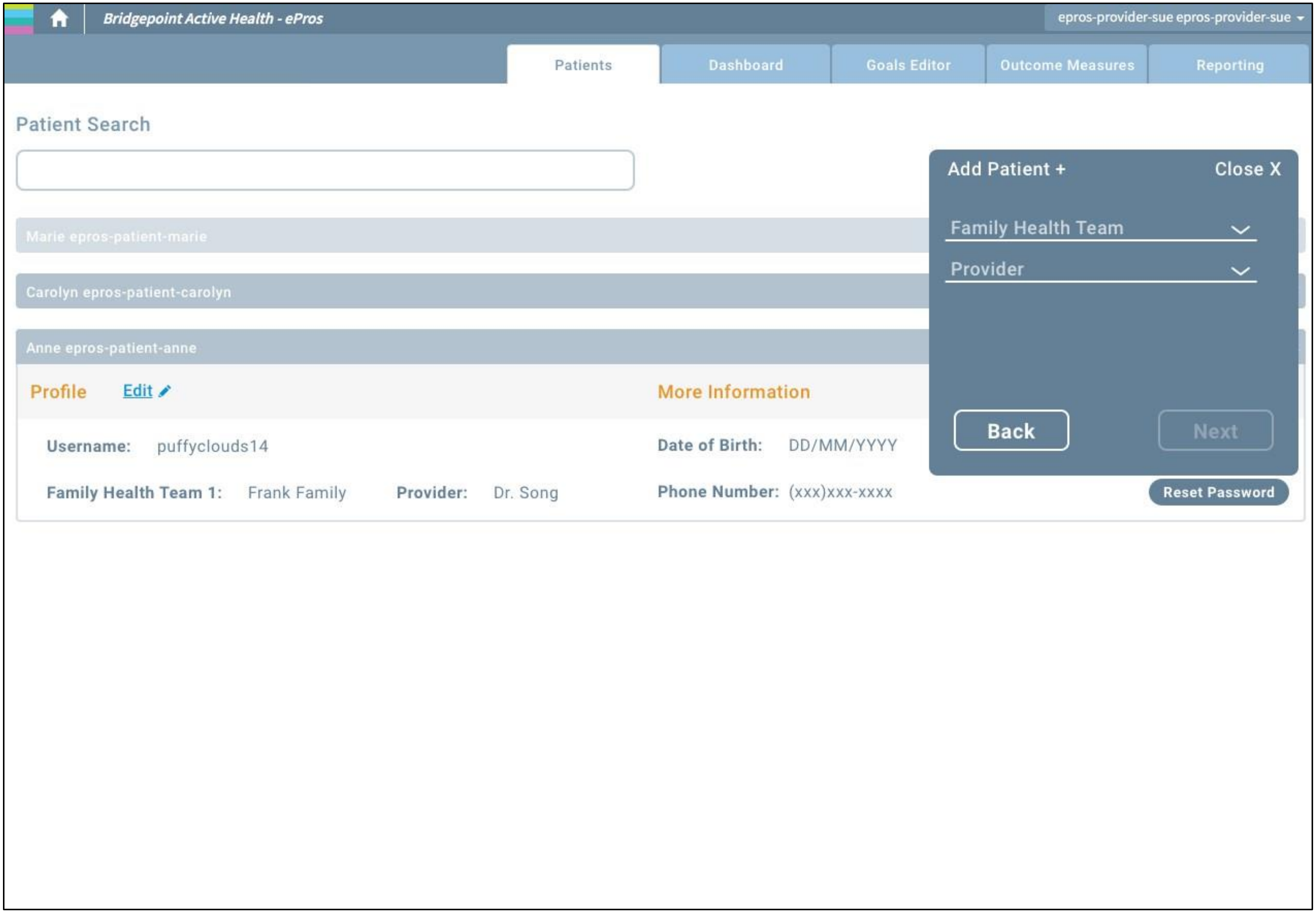
Bridgepoint Active Health - ePros
epros-provider-sue epros-provider-sue
Patients
Dashboard
Goals Editor
Outcome Measures
Reporting
Patient Search
Marie epros-patient-marie
Carolyn epros-patient-carolyn
Anne epros-patient-anne
Profile
Edit
More Information
Username: puffyclouds14
Date of Birth: DD/MM/YYYY
Family Health Team 1: Frank Family
Provider: Dr. Song
Phone Number: (xxx)xxx-xxxx
Reset Password
Add Patient +
Close X
Family Health Team
Provider
Back
Next

Setting goals

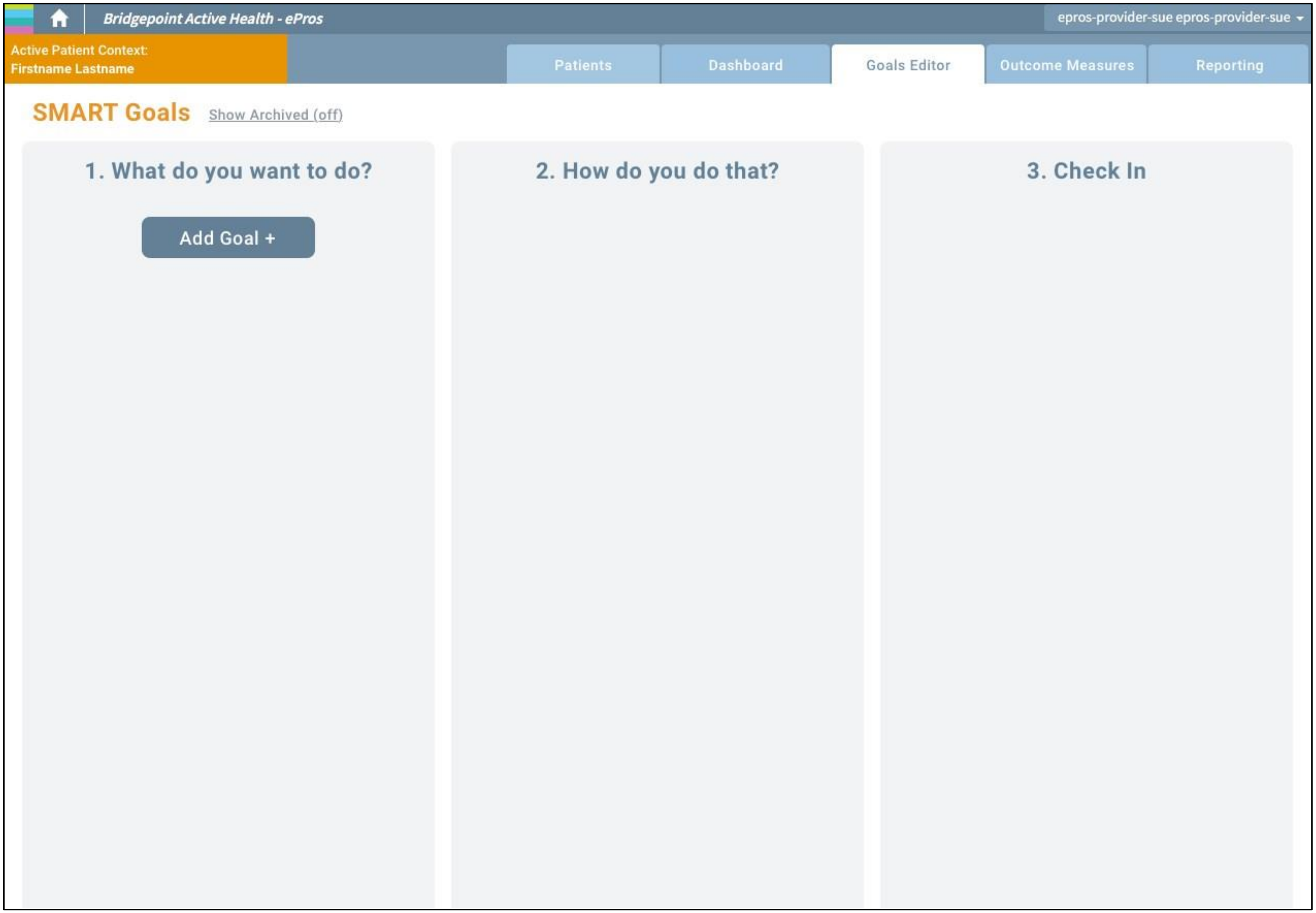

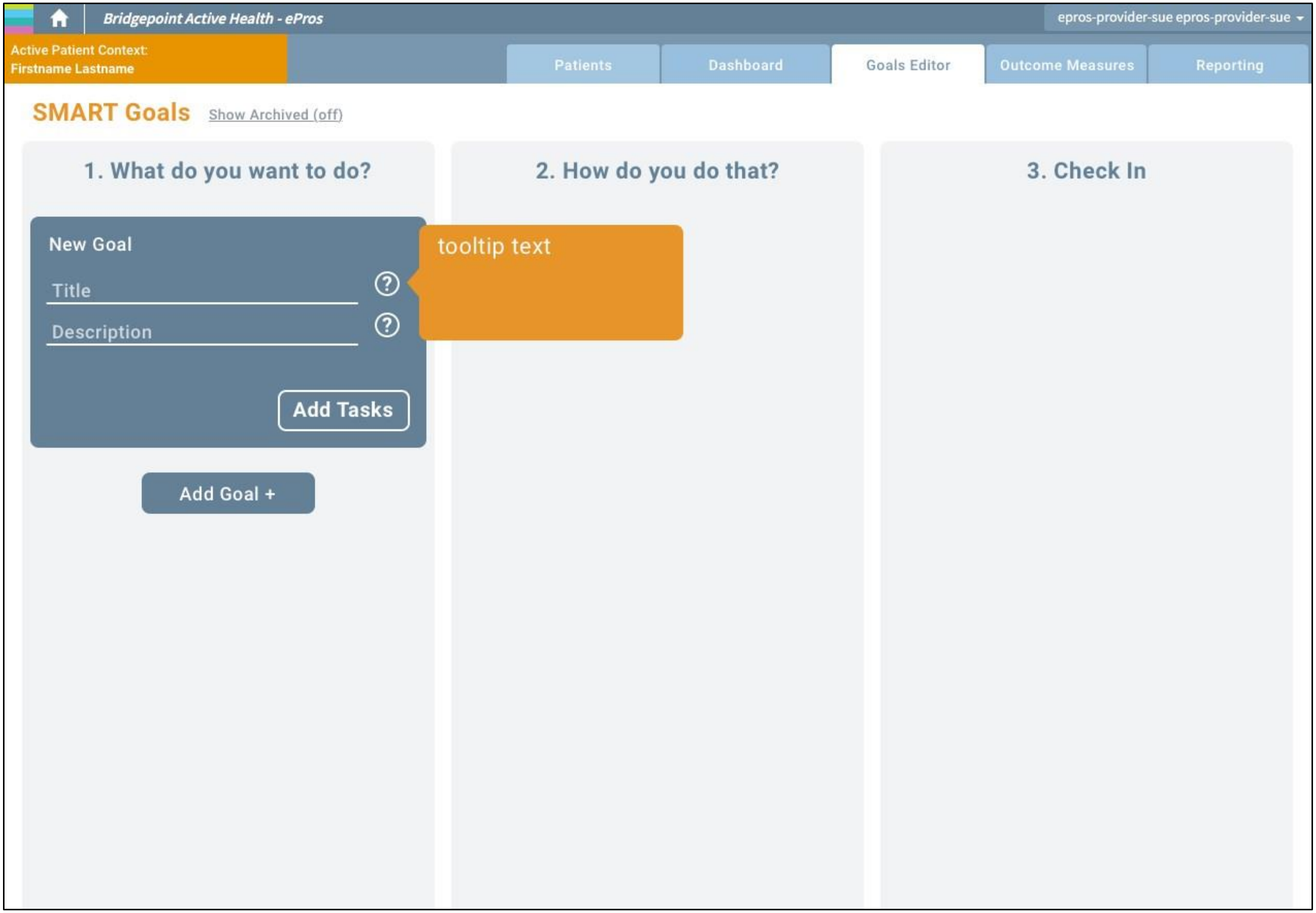
Bridgepoint Active Health - ePros
epros-provider-sue epros-provider-sue
Active Patient Context:
Firstname Lastname
Patients
Dashboard
Goals Editor
Outcome Measures
Reporting
SMART Goals
Show Archived (off)
1. What do you want to do?
2. How do you do that?
3. Check In
New Goal
Title
Description
tooltip text
Add Tasks
Add Goal +

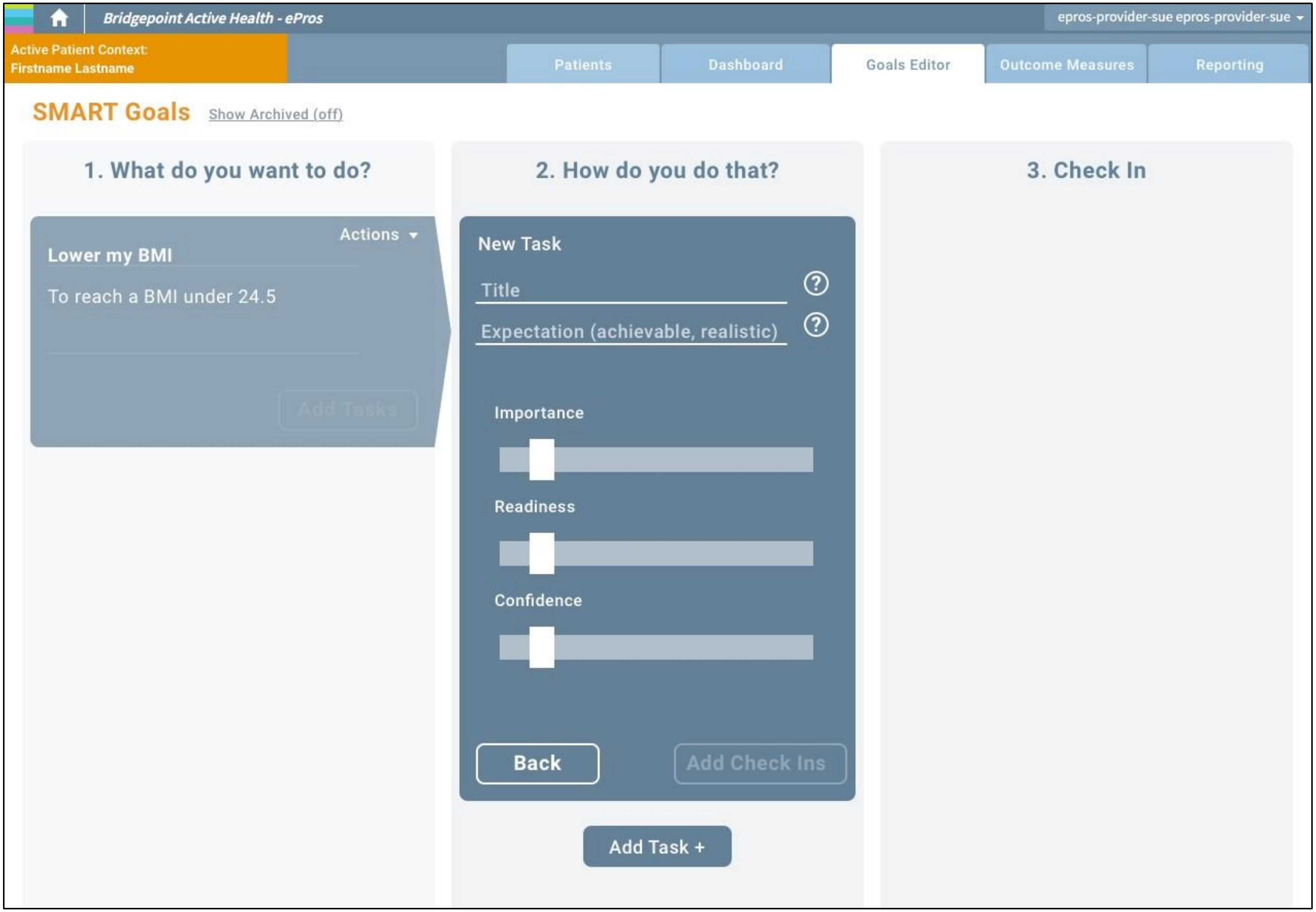
Bridgepoint Active Health - ePros
epros-provider-sue epros-provider-sue
Active Patient Context:
Firstname Lastname
Patients
Dashboard
Goals Editor
Outcome Measures
Reporting
SMART Goals
Show Archived (off)
1. What do you want to do?
2. How do you do that?
3. Check In
Actions
Lower my BMI
To reach a BMI under 24.5
Add Tasks
New Task
Title
Expectation (achievable, realistic)
Importance
Readiness
Confidence
Back
Add Check Ins
Add Task +

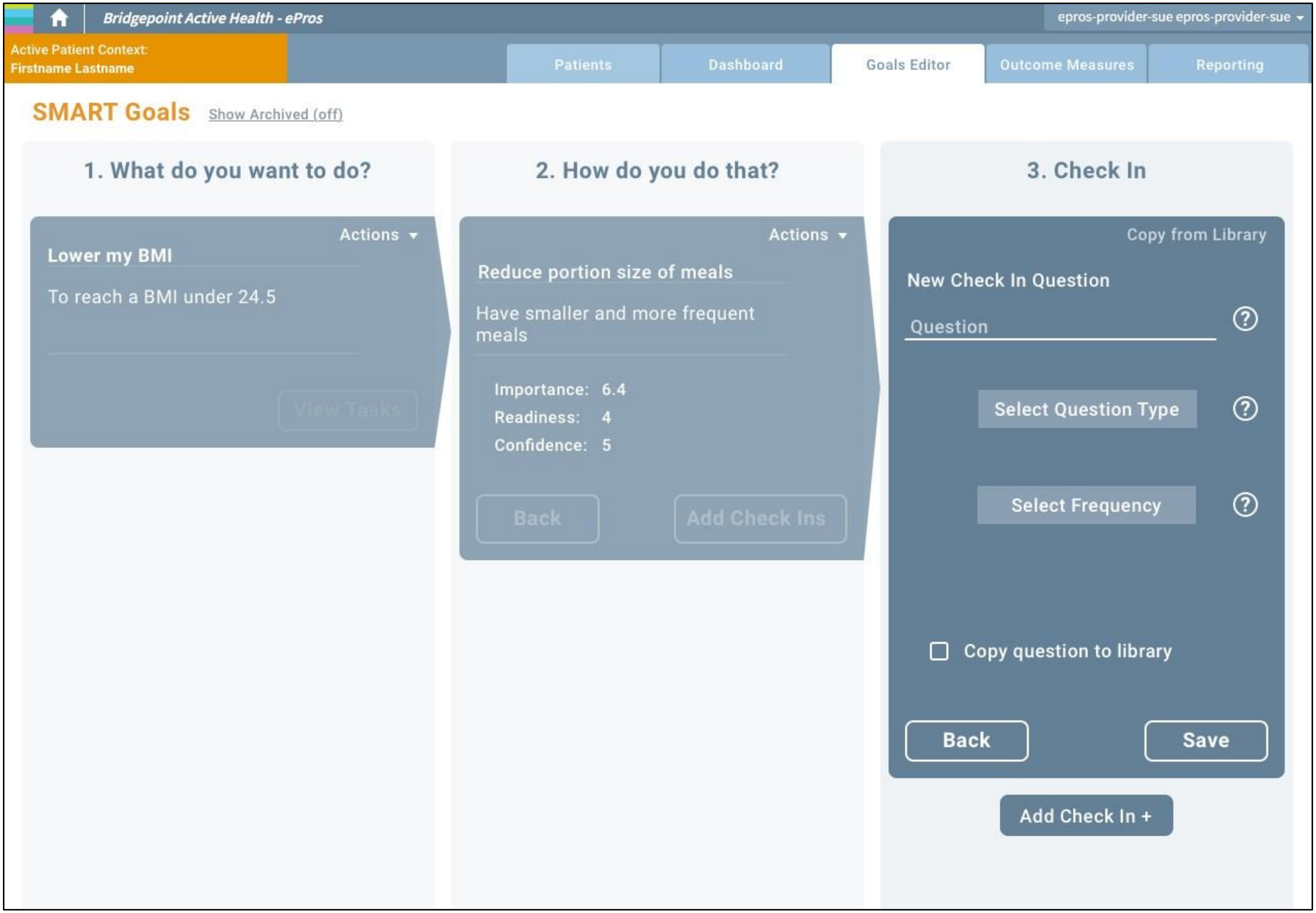
Bridgepoint Active Health - ePros
epros-provider-sue epros-provider-sue
Active Patient Context:
Firstname Lastname
Patients
Dashboard
Goals Editor
Outcome Measures
Reporting
SMART Goals
Show Archived (off)
1. What do you want to do?
Actions
Lower my BMI
To reach a BMI under 24.5
View Tasks
2. How do you do that?
Actions
Reduce portion size of meals
Have smaller and more frequent meals
Importance: 6.4
Readiness: 4
Confidence: 5
Back
Add Check Ins
3. Check In
Copy from Library
New Check In Question
Question
Select Question Type
Select Frequency
Copy question to library
Back
Save
Add Check In +

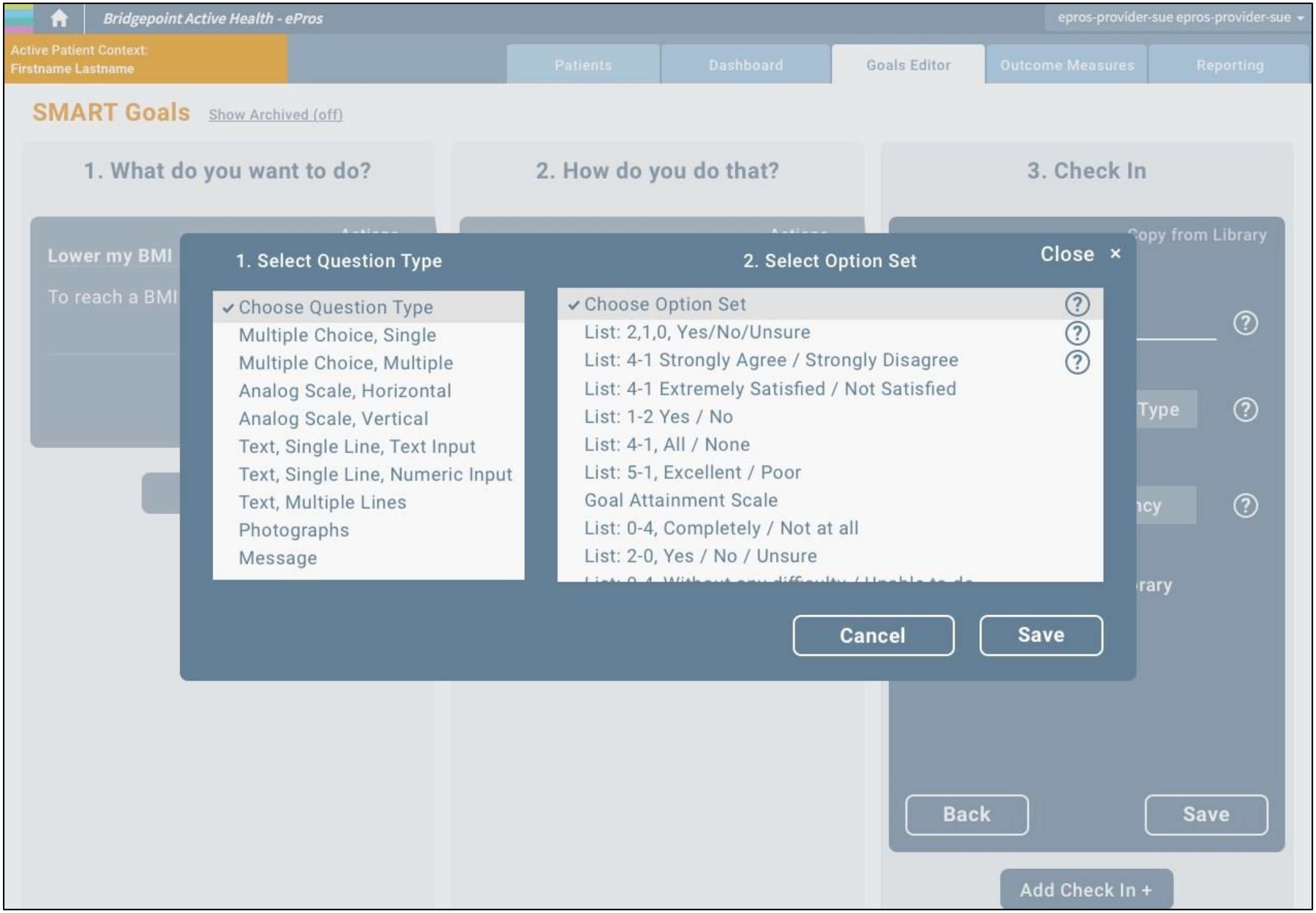
Bridgepoint Active Health - ePros
epros-provider-sue epros-provider-sue
Active Patient Context:
Firstname Lastname
Patients
Dashboard
Goals Editor
Outcome Measures
Reporting
SMART Goals
Show Archived (off)
1. What do you want to do?
2. How do you do that?
3. Check In
Lower my BMI
To reach a BMI
Copy from Library
1. Select Question Type
2. Select Option Set
Close ×
Choose Question Type
Multiple Choice, Single
Multiple Choice, Multiple
Analog Scale, Horizontal
Analog Scale, Vertical
Text, Single Line, Text Input
Text, Single Line, Numeric Input
Text, Multiple Lines
Photographs
Message
Choose Option Set
List: 2,1,0, Yes/No/Unsure
List: 4-1 Strongly Agree / Strongly Disagree
List: 4-1 Extremely Satisfied / Not Satisfied
List: 1-2 Yes / No
List: 4-1, All / None
List: 5-1, Excellent / Poor
Goal Attainment Scale
List: 0-4, Completely / Not at all
List: 2-0, Yes / No / Unsure
Cancel
Save
Back
Save
Add Check In +

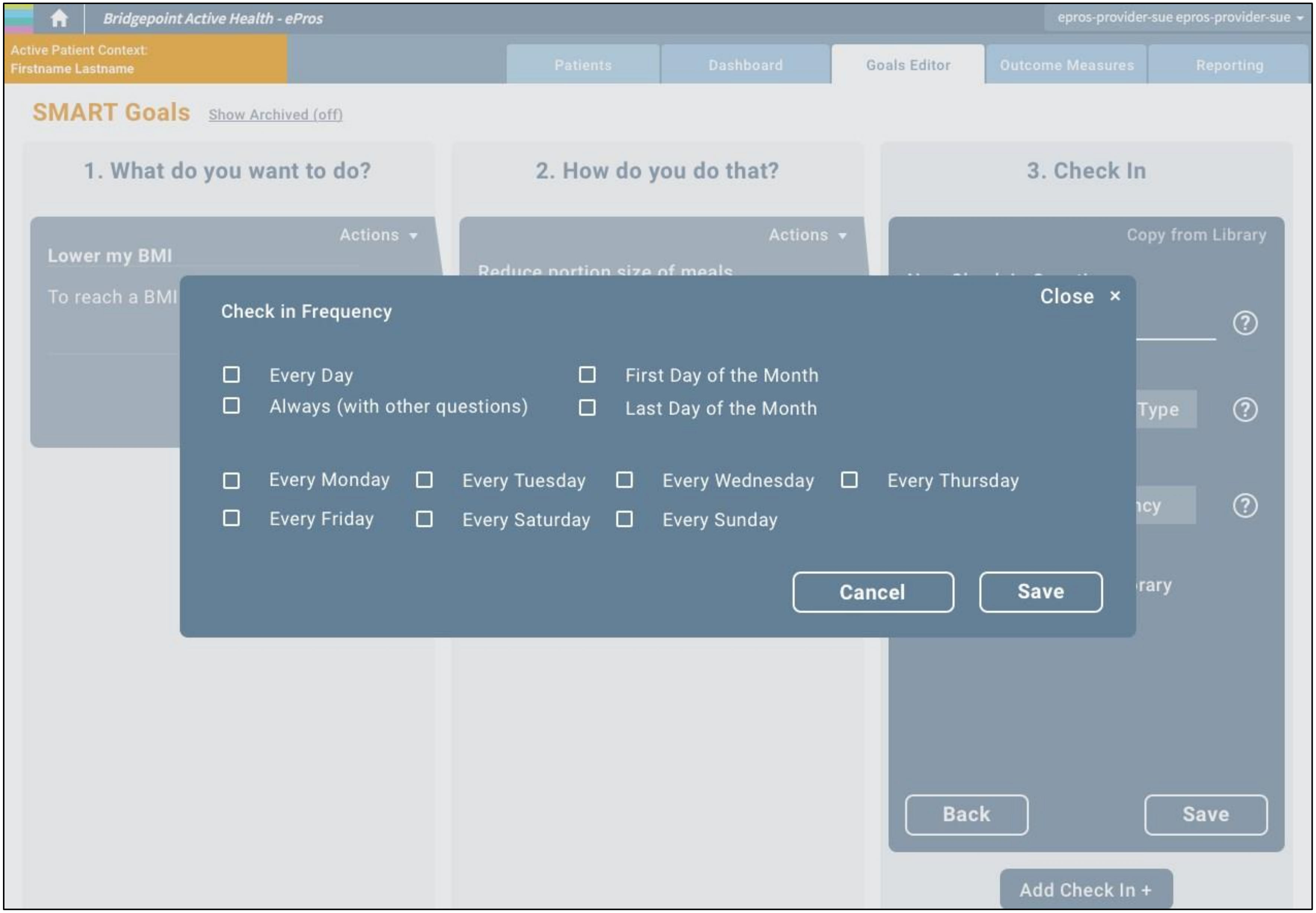
Bridgepoint Active Health - ePros
epros-provider-sue epros-provider-sue
Active Patient Context:
Firstname Lastname
Patients
Dashboard
Goals Editor
Outcome Measures
Reporting
SMART Goals
Show Archived (off)
1. What do you want to do?
2. How do you do that?
3. Check In
Actions
Lower my BMI
To reach a BMI
Copy from Library
Check in Frequency
Close ×
Every Day
Always (with other questions)
First Day of the Month
Last Day of the Month
Every Monday
Every Tuesday
Every Wednesday
Every Thursday
Every Friday
Every Saturday
Every Sunday
Cancel
Save
Back
Save
Add Check In +

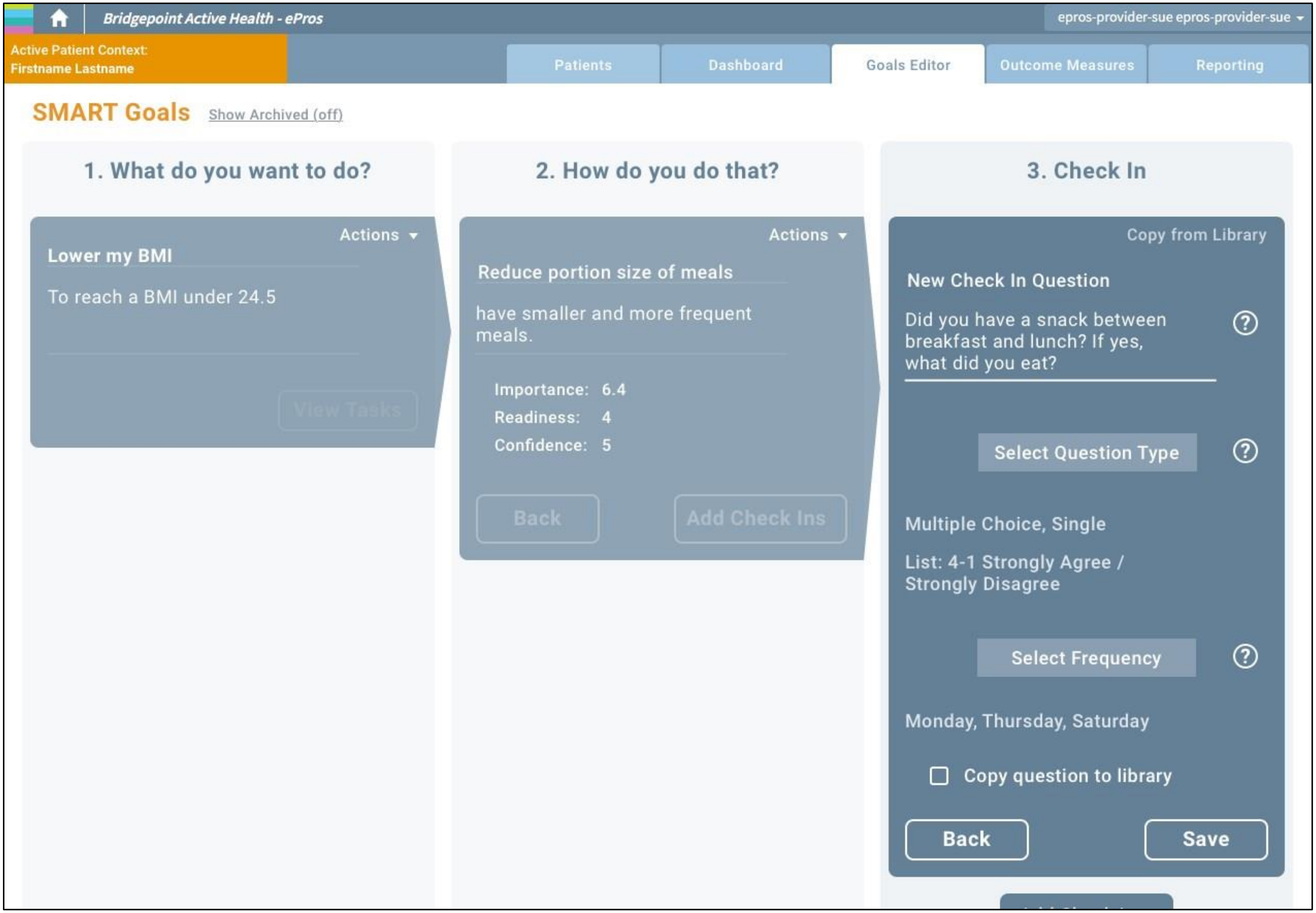
Bridgepoint Active Health - ePros
epros-provider-sue epros-provider-sue
Active Patient Context:
Firstname Lastname
Patients
Dashboard
Goals Editor
Outcome Measures
Reporting
SMART Goals
Show Archived (off)
1. What do you want to do?
Actions
Lower my BMI
To reach a BMI under 24.5
View Tasks
2. How do you do that?
Actions
Reduce portion size of meals
have smaller and more frequent meals.
Importance: 6.4
Readiness: 4
Confidence: 5
Back
Add Check Ins
3. Check In
Copy from Library
New Check In Question
Did you have a snack between breakfast and lunch? If yes, what did you eat?
Select Question Type
Multiple Choice, Single
List: 4-1 Strongly Agree / Strongly Disagree
Select Frequency
Monday, Thursday, Saturday
Copy question to library
Back
Save

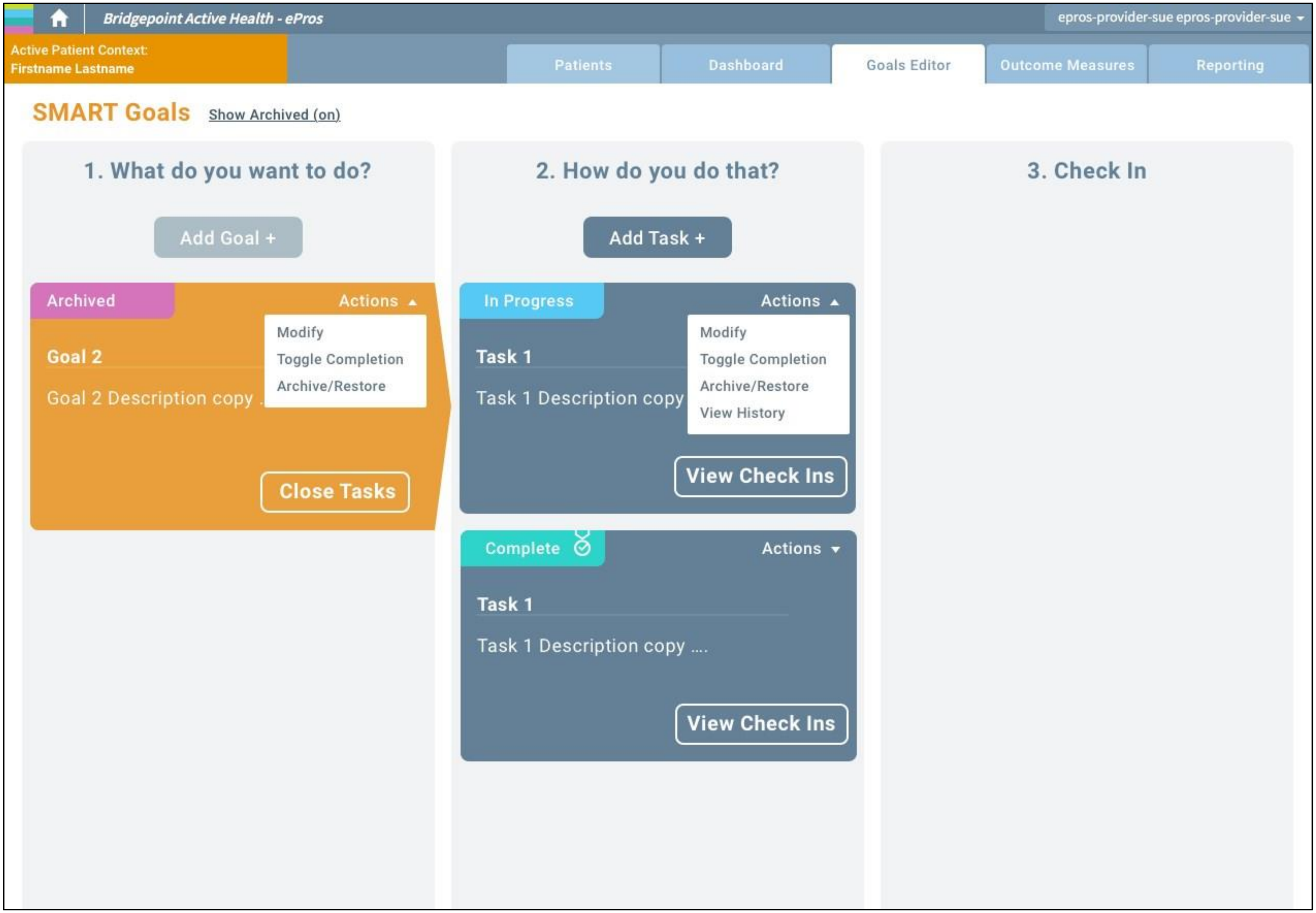

Bridgepoint Active Health - ePros
epros-provider-sue epros-provider-sue
Active Patient Context:
Firstname Lastname
Patients
Dashboard
Goals Editor
Outcome Measures
Reporting
SMART Goals
Show Archived (on)
1. What do you want to do?
Add Goal +
Archived
Actions
Modify
Toggle Completion
Archive/Restore
Goal 2
Goal 2 Description copy
Close Tasks
2. How do you do that?
Add Task +
In Progress
Actions
Modify
Toggle Completion
Archive/Restore
View History
Task 1
Task 1 Description copy
View Check Ins
Complete
Actions
Task 1
Task 1 Description copy ....
View Check Ins
3. Check In

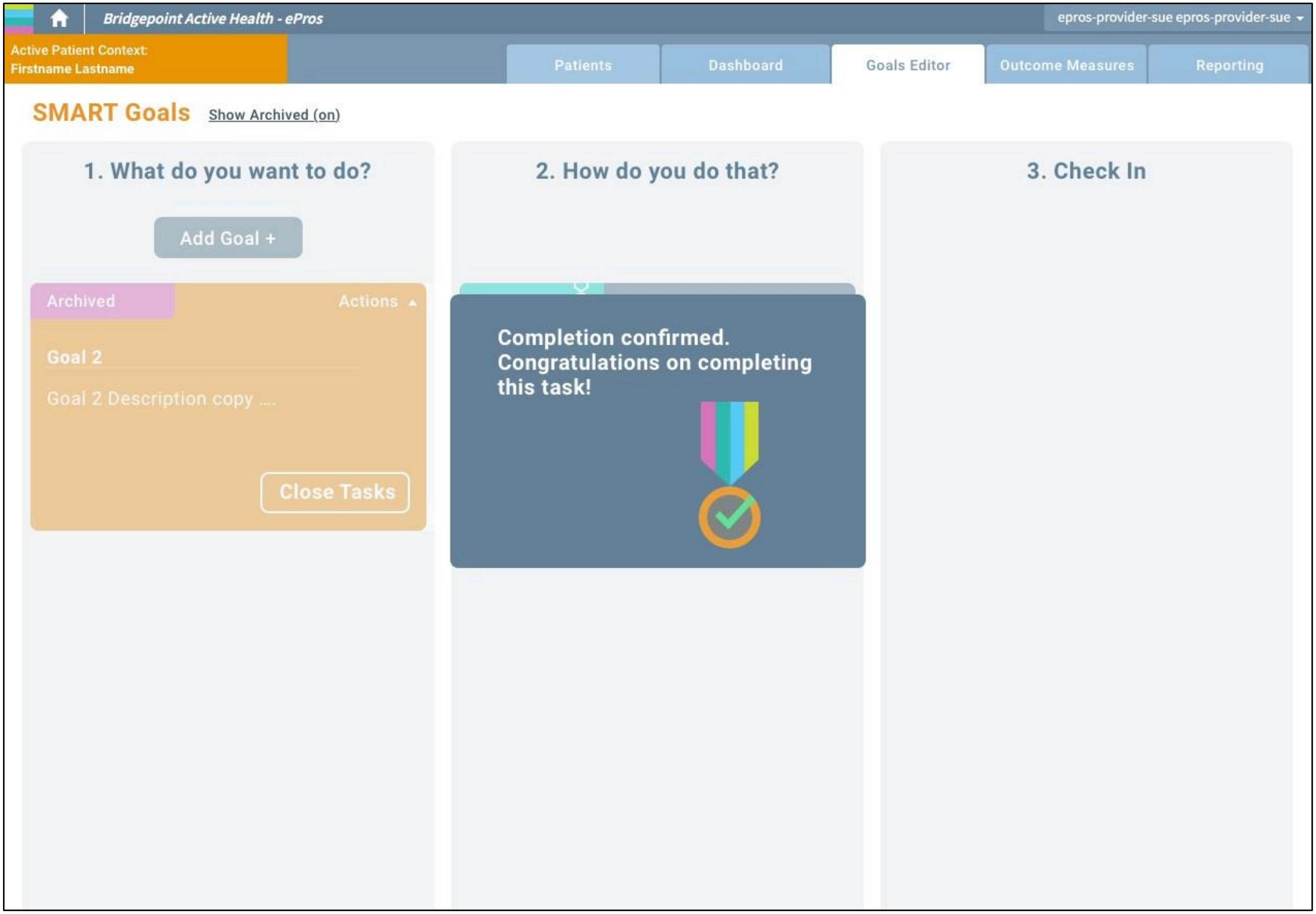
Bridgepoint Active Health - ePros
epros-provider-sue epros-provider-sue
Active Patient Context:
Firstname Lastname
Patients
Dashboard
Goals Editor
Outcome Measures
Reporting
SMART Goals
Show Archived (on)
1. What do you want to do?
Add Goal +
Archived
Actions
Goal 2
Goal 2 Description copy ....
Close Tasks
2. How do you do that?
Completion confirmed. Congratulations on completing this task!
3. Check In

Project:	Bridgepoint ePros
Layout	Android Splash Screen

BRIDGEPOINT
active healthcare

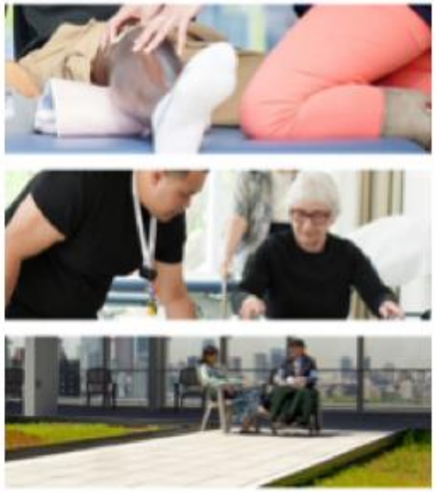

Privacy	This Document Is Confidential

Project:	Bridgepoint ePros
Layout	Home Dashboard

12:00

My Goals

Maintain Independence 4

Lorem ipsum dolor sit amet, consectetur adipiscing elit. Ut pharetra pretium enim sed accumsan. Curabitur tristique, lacus at viverra accumsan, lacus lectus bibendum nibh, nec ultricies risus eros eget nibh.

Feedback reminder, tap here to complete your feedback for this goal.

Other Goal

Lorem ipsum dolor sit amet, consectetur adipiscing elit. Ut pharetra pretium enim sed accumsan. Curabitur tristique, lacus at viverra accumsan, lacus lectus bibendum nibh, nec ultricies risus eros eget nibh.

Privacy	This Document Is Confidential

Project:	Bridgepoint ePros
Layout	Feedback Home

12:00

Maintain Independence

Feedback | Charts | Photos

Today 4

There are 12 active questions for:
Goal: Walk More
Goal: Dress Self

So far you have answered 8 of the required questions. There are also 3 optional questions.

Yesterday ✓

There were 4 active questions for:
Goal: Walk More

So far you have answered 4 of the required questions.

Privacy	This Document Is Confidential

Project:	Bridgepoint ePros
Layout	Feedback Survey A

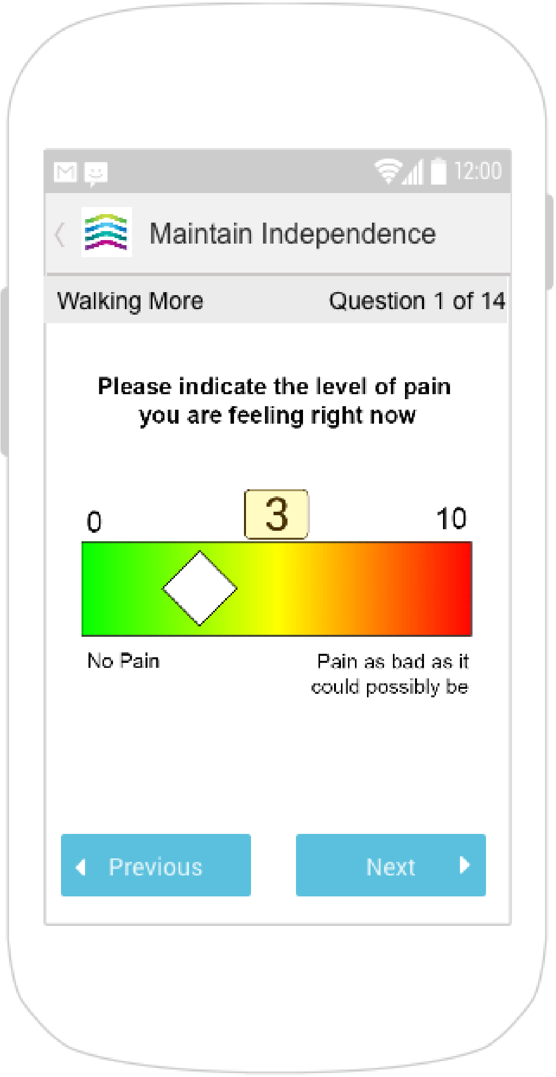

Privacy	This Document Is Confidential

Project:	Bridgepoint ePros
Layout	Feedback Survey B

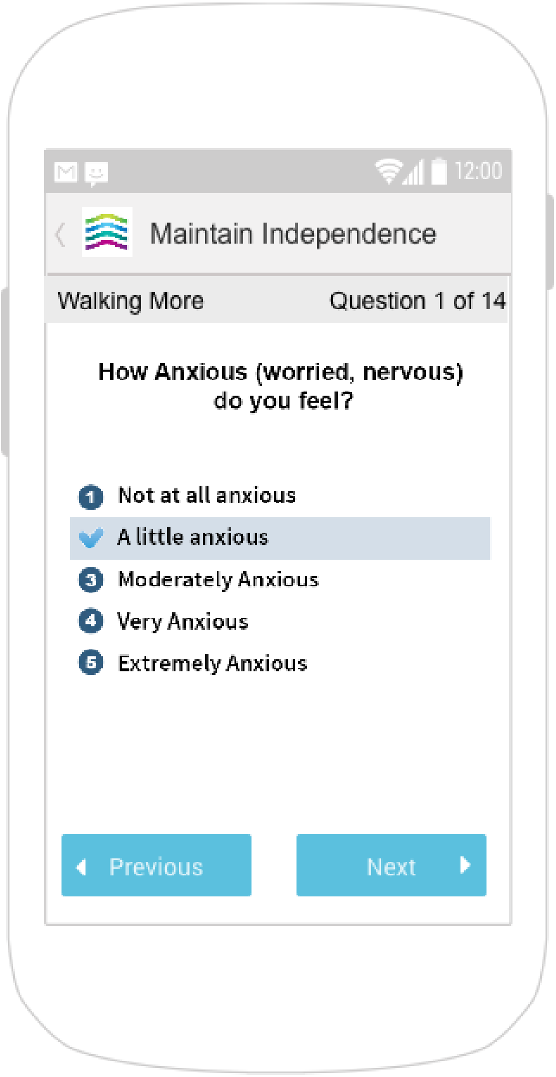

Privacy	This Document Is Confidential

Project:	Bridgepoint ePros
Layout	Charts / Results / Graphs

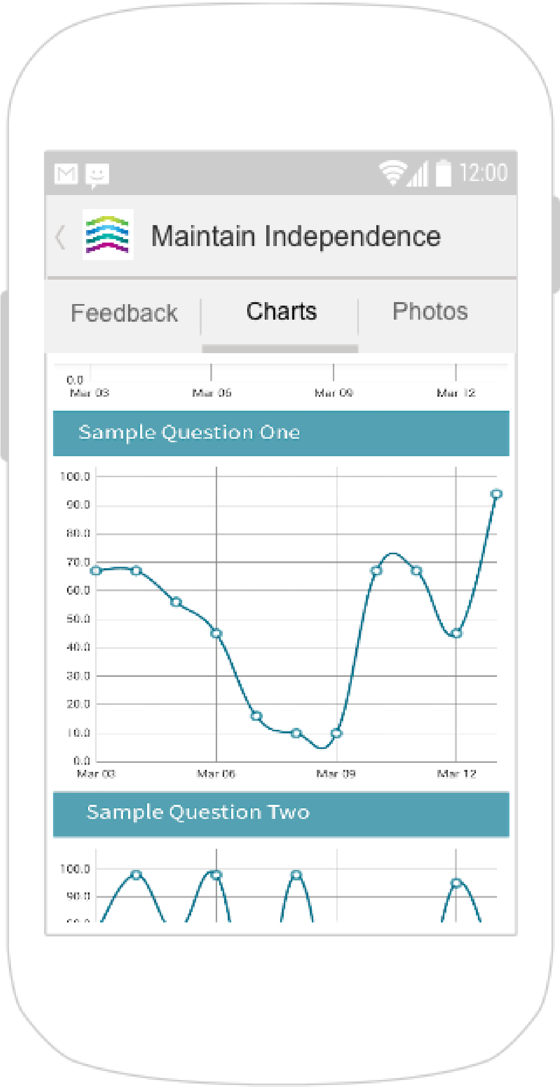

Privacy	This Document Is Confidential
