# Supplementary material for: Building a Digital Bridge to Support Patient-Centered Care Transitions From Hospital to Home for Older Adults With Complex Care Needs: Protocol for a Co-Design, Implementation, and Evaluation Study
Source: JMIR Res Protoc. 2020 Nov 25;9(11):e20220. doi: 10.2196/20220 (PMC7725647; doi:10.2196/20220)
Supplement: Multimedia Appendix 3 [file resprot_v9i11e20220_app3.docx]

## Multimedia Appendix 3: Description of Digital Bridge Workflow

### Step 1: Patient onboarding, in-hospital care and connecting to primary care

Patients selected to join the study will be identified at the time of admission to service units where Care Connector is in use, and will be given electronic access to the technology. Studies of patient portal use by hospitalized patients suggest good patient enrollment rates[1]. Clinicians on participating services will use Care Connector to coordinate patient care in-hospital, enabling patient adoption and practice integration. When patients join the study, the research team will connect with the patient’s PCP, a feasible approach we have used in other care transition models[2]. If the PCP is agreeable, hospital clinicians may also communicate with them using Care Connector while patient is in hospital. In rehabilitation service, patients and clinicians will also use ePRO to support goal-oriented care practices during their 4-6 week in-hospital stay.

### Step 2: Discharge and transition to primary care team

Patients and family caregivers will work with their team to engage in discharge planning from the moment of admission as aligned with suggested best practice in transition planning. At the appropriate time point, the electronic tool will be introduced (the co-design process will help determine at which point this is likely to occur). Once the PODS is created, the patient and hospital provider will be prompted to set transition goals using the ePRO tool. We anticipate some goals may reflect elements of the discharge plan to facilitate successful transitions (e.g. heart failure patients may set a goal of measuring weight daily after discharge to monitor for signs of volume overload). Patients can use their mobile device or home computer to track transition related goals. The official discharge summary and PODS will be sent to the PCP, along with a link to the ePRO tool so they can connect to the patient goals. Systems do not currently integrate into primary care medical records; feasibility and desirability for this will be explored in the co-design process. Implementation of the ePRO tool has been successful as a stand-alone tool.

### Step 3: 7-day follow-up

Patients discharged from either setting will connect with their PCP within 7 days post-discharge as aligned with standard practice. PCP will then have both PODS and ePRO goals available to guide this conversation. PCP and patients will have the option of modifying or setting new goals.

### Step 4: Maintenance in the community setting

Following the current ePRO process being implemented in 6 FHTs across Ontario, patients will use ePRO to self-monitor and manage their health needs. PCP can choose to continue to engage with the tool, as well to help tailor patient care to person-centred goals.

1. Dalal, A.K., et al., *A web-based, patient-centered toolkit to engage patients and caregivers in the acute care setting: A preliminary evaluation.* Journal of the American Medical Informatics Association, 2015. **23**(1): p. 80-87.

2. Bansal, P., et al. *From hospital to home...and back again? Stories from the bridge to home team on improving discharges for complex patients*. in *GTA Rehab Network*. 2015.
