# Supplementary material for: Building a Digital Bridge to Support Patient-Centered Care Transitions From Hospital to Home for Older Adults With Complex Care Needs: Protocol for a Co-Design, Implementation, and Evaluation Study
Source: JMIR Res Protoc. 2020 Nov 25;9(11):e20220. doi: 10.2196/20220 (PMC7725647; doi:10.2196/20220)
Supplement: Multimedia Appendix 4 [file resprot_v9i11e20220_app4.pdf]

Multimedia Appendix 4: Grant Reviewer Reports

Review Type/Type d'évaluation: Committee Member 1/Membre de comité 1
Name of Applicant/Nom du chercheur: Steele Gray, Carolyn Alice
Application No./Numéro de demande: 418448
Agency/Agence: CIHR/IRSC
Competition/Concours: 2019-01-24 Team Grant: Transitions in Care/Subvention d'équipe : Transitions dans les soins
Committee/Comité: Team Grant: Transitions in Care/Subvention d'équipe : Transitions dans les soins
Title/Titre: The Digital Bridge: Using technology to support patient-centred transitions from hospital to home

---

Assessment/Évaluation:

Rationale and Research Question

The rationale for the work is that older adults with complex care needs are high-cost and frequent users of the healthcare system, suggesting that improving transitions between home and hospital will lead to improved healthcare delivery and health-related outcomes. This rationale is sound and fits within the funding opportunity. More specifically, the applicants note that poor communication during the transition process, phenomenon such as “post-hospital syndrome”, and patients’ limited ability to adjust to the transition, all lead to poor patient outcomes and readmission. They also note that digital solutions, and particularly information systems and multi-professional care coordination, create high user satisfaction and promote quality of life for older adults. Overall, the applicants propose that “communication that enables person-centred care and self-management may offer the greatest advantages as we support older adults with CCN transitioning from hospital to home”.

The proposed research questions are considerate and fall nicely within the objectives of the funding opportunity. Specifically, the proposed work will improve health outcomes, patient experience and lower the cost of healthcare through a scalable solution, built by engaging multiple partners.

Planned Intervention/Solution(s) and Research Methodology

The planned intervention is a digital bridge that will provide support from the point of hospitalization, through discharge, and once in the community again. This service will be accessible by hospital providers, personal care providers and patients. It will integrate two existing systems (Care Connector and electronic Patient Reported Outcomes). Care Connector provides communication and collaboration features, focused on discharge planning, but does not provide ongoing support after transition. ePRO provides a goal tracker and a health journal. The intervention novelty is minimal, considering both Care Connector and ePRO already exist and are available. Indeed, the contribution is, as noted in the research questions, almost entirely process-related in terms of identifying the workflow considerations and the economic benefits of the system.

Methodologically, the study will be comprised of three phases. In Phase 1, an unspecified number of working groups will contribute to identifying the transition workflow. No detail is given or hypotheses presented on what this workflow may look like, substantiating the likelihood of being able to adapt the existing Care Connector and ePRO technologies in the necessary way. As such, it is not clear whether this will even be possible. In Phase 2, an iterative implementation and redesign approach is proposed based on an unspecified pragmatic before-after study design. Methodological details are sparse here (e.g., study design, data collection points, full sample size calculations, justification for secondary outcomes, and more relevant primary outcome(s)). Phase 3 focuses on KT and scale and spread strategy development. The outcome measures are primitive, and little attention is given to the analyses that will be used to process the data.

Team

Review Type/Type d'évaluation: Committee Member 1/Membre de comité 1
Name of Applicant/Nom du chercheur: Steele Gray, Carolyn Alice
Application No./Numéro de demande: 418448
Agency/Agence: CIHR/IRSC
Competition/Concours: 2019-01-24 Team Grant: Transitions in Care/Subvention d'équipe : Transitions dans les soins
Committee/Comité: Team Grant: Transitions in Care/Subvention d'équipe : Transitions dans les soins
Title/Titre: The Digital Bridge: Using technology to support patient-centred transitions from hospital to home

---

Assessment/Évaluation:

The research team is well-suited to carry out the proposed research. Specifically, the two project leads for the existing solutions (Care Connector and ePRO) are co-principal applicants on the project. The team's partnership with Canada Health Infoway also helps bring expertise in data collection and data understanding in the context of the project. The team also has a good breadth of scientific, clinical and patient representatives. However, the team is notably missing a member with data analysis background.

Capacity Building & Environment

The team's research program offers minimal engagement with trainees and/or new researchers. Most of the personnel funding is for professional staff, though marginal funding is allocated to support a post-doctoral fellow (Y2-4) and a graduate student (Y3-4). The environment, however, is supportive of the proposed activities, and implementation support is noted as confirmed at both sites. Accordingly, there should be no issues with access to personnel, facilities and infrastructure.

Viability of the eHealth innovation partner(s) (Stream 2 only)

Not applicable.

Review Type/Type d'évaluation: Committee Member 2/Membre de comité 2
Name of Applicant/Nom du chercheur: Steele Gray, Carolyn Alice
Application No./Numéro de demande: 418448
Agency/Agence: CIHR/IRSC
Competition/Concours: 2019-01-24 Team Grant: Transitions in Care/Subvention d'équipe : Transitions dans les soins
Committee/Comité: Team Grant: Transitions in Care/Subvention d'équipe : Transitions dans les soins
Title/Titre: The Digital Bridge: Using technology to support patient-centred transitions from hospital to home

---

Assessment/Évaluation:

Summary: This project will address the gaps in TiC for elderly patients by implementing and evaluating a Digital Bridge to support person-centred health care transitions for older adults with complex care needs. The Digital Bridge will integrate an existing hospital-based communication tool (Care Connector) with a community-based patient-goal setting tool (the electronic Patient Reported Outcomes (ePRO) tool) to support person-centred communication through the entire transition pathway starting in the hospital through 6 months post-discharge. The project poses three research questions regarding: 1) the most appropriate workflow design of the digital model; 2) the cost-effectiveness and impact of the model; and 3) implementation barriers and enablers.

Strengths

1- strong justification for shared situational awareness in the potential impact of efficient IT solutions to improve better communication in support of TiC
2- integration of hospital based and primary care based digital health technologies
3- strong co-design methodology
4- measurement of the relational coordination
5- the economic evaluation is well described
6- qualitative methodology well planned and described
7- strong team track record with previous experience in running complex interventions
8- strong partnerships with Canada Health Infoway and QoC Health Inc.
9- capacity building plan includes graduate students and post-doc
10- significant cash contributions from partners: QoC, Sinai, and Trillium
11- strong publication record by research team

Weaknesses

1- Why isn't the full patient medical record included in the Digital Bridge?
2- before and after design is not clearly described
3- before and after design will be prone to bias and potentially will not give a valid estimation of cost-analysis
4- Although the CTM3 is a validated and a frequently used tool, the application lacks detail about limitations with use of the CTM3 score: eg: how to deal with patients who can't answer questions? missing data? What is the rate of expected missing data? The potential risks to this study and the mitigation strategies used are not sufficiently described.

**Review Type/Type d'évaluation:**	Committee Member 3/Membre de comité 3
**Name of Applicant/Nom du chercheur:**	Steele Gray, Carolyn Alice
**Application No./Numéro de demande:**	418448
**Agency/Agence:**	CIHR/IRSC
**Competition/Concours:**	2019-01-24 Team Grant: Transitions in Care/Subvention d'équipe : Transitions dans les soins
**Committee/Comité:**	Team Grant: Transitions in Care/Subvention d'équipe : Transitions dans les soins
**Title/Titre:**	The Digital Bridge: Using technology to support patient-centred transitions from hospital to home

Assessment/Évaluation:

Rationale and Research Question

STRENGTHS

Older adults with multi-morbidity and complex care needs (CCN) are among those most likely to experience frequent care transitions from hospital to home, and many fall into the category of high-cost users, accounting for the majority of year-over-year healthcare spending in Ontario, Canada and internationally. The complexity these individuals stems not only from their multi-morbidity disease profiles, but also the social, environmental and contextual issues that make it difficult for them to manage their physical health needs. The interaction of these challenges often results in frequent hospital admissions. The transition from hospital to home is often challenging leading to poor patient outcomes and higher rates of readmission. Improved communication that enables person-centred care may offer the greatest advantages to support older adults with complex care needs transitioning from hospital to home.

Digital health technologies offer a promising solution to support person-centred communication across inter professional teams working within and between health care organizations. A systematic review of inter-professional communication in transitional care models found that information systems, as well as multi-professional care coordination, support higher satisfaction and subjective quality of life for older adults. A key strength of digital solutions is their ability to foster shared situational awareness of interprofessional teams. However, existing solutions rarely span boundaries, are not sufficiently person-centred, and lack a robust evidence-base. majority of communication systems exist within single teams, few are co-designed with patients and providers, only been evaluated over short periods with insufficient attention to implementation as a means to support both evidence of effectiveness as well as transferability of findings

The project will address these gaps by implementing and evaluating Digital Bridge to support person-centred health

**Review Type/Type d'évaluation:**	Committee Member 3/Membre de comité 3
**Name of Applicant/Nom du chercheur:**	Steele Gray, Carolyn Alice
**Application No./Numéro de demande:**	418448
**Agency/Agence:**	CIHR/IRSC
**Competition/Concours:**	2019-01-24 Team Grant: Transitions in Care/Subvention d'équipe : Transitions dans les soins
**Committee/Comité:**	Team Grant: Transitions in Care/Subvention d'équipe : Transitions dans les soins
**Title/Titre:**	The Digital Bridge: Using technology to support patient-centred transitions from hospital to home

Assessment/Évaluation:

care transitions for older adults with complex care needs. Digital Bridge will integrate an existing hospital-based communication tool (Care Connector) with a community-based patient-goal setting tool (the electronic Patient Reported Outcomes (ePRO) tool) to support person-centred communication through the entire transition pathway starting in the hospital through 6 months post-discharge. The project poses three research questions regarding: 1) the most appropriate workflow design of the digital model; 2) the cost-effectiveness and impact of the model; and 3) implementation barriers and enablers.

This project, advancing a person-centred transition model of care, will be among the first to integrate hospital based and community based digital health technologies for older adults with complex care needs. With improved communication around transitions several down-stream outcomes with wider adoption may be affected, including: improved patient experience, greater efficiency and care coordination, and reduced unnecessary readmission and emergency department use for patients post-discharge leading to cost savings.

The team will examine the impact of socio-demographic variables, including sex, ethnicity, as well as socio-economic status in the analysis. Sex and gender differences in health and functional status, socio-economic circumstances, and social roles (i.e. caregiver roles responsibilities) are commonly noted in the literature and may be particularly relevant for older adults with women taking on substantial caregiving activities. Women additionally have different health care seeking and survival patterns as compared to males. Patient socioeconomic status and ethnicity are also key demographic variables that can impact on patient outcomes. cognizant that those who are more deprived, and have less education and literacy skills may be at a greater disadvantage to using and accepting the type of technology being tested in study, potentially widening an existing health disparity between the social classes. Technology seeking behaviours may also differ across gender groups which will be explored. We anticipate that these demographic factors will all have an impact on patient health outcomes and as such will be explored as part of the evaluation of the Digital Bridge. By including this analysis, the study will contribute to a better understanding of patient needs and their contexts, and how this may affect the adoption of technologies as part of health care service delivery. The will additionally explore these gender differences across provider participants, as use of technology is tied to social norms which may be grounded in gender roles as well

**Review Type/Type d'évaluation:**	Committee Member 3/Membre de comité 3
**Name of Applicant/Nom du chercheur:**	Steele Gray, Carolyn Alice
**Application No./Numéro de demande:**	418448
**Agency/Agence:**	CIHR/IRSC
**Competition/Concours:**	2019-01-24 Team Grant: Transitions in Care/Subvention d'équipe : Transitions dans les soins
**Committee/Comité:**	Team Grant: Transitions in Care/Subvention d'équipe : Transitions dans les soins
**Title/Titre:**	The Digital Bridge: Using technology to support patient-centred transitions from hospital to home

Assessment/Évaluation:

The application is responsive to the call for research that transforms the health system to optimize the outcomes of patients experiencing transitions in care. Appropriate and expert aging and socio-demographic variables, including sex, ethnicity, as well as socio-economic status are planned.

WEAKNESSES

None

Planned Intervention/Solution(s) and Research Methodology

STRENGTHS

The proposal appropriately identifies the population challenges, gaps and inefficiencies that impact TiC, as well as an intervention and solution. They are clear in their rationale, proposed solution and research methodology. Outcome measures to patients are presented. Very high quality approach to implementation, and evaluation including cost effectiveness are presented. The budget is appropriate to support the activities. There is adequate plant for adapting, sustaining and scaling the proposed intervention beyond the grant.

**Review Type/Type d'évaluation:**	Committee Member 3/Membre de comité 3
**Name of Applicant/Nom du chercheur:**	Steele Gray, Carolyn Alice
**Application No./Numéro de demande:**	418448
**Agency/Agence:**	CIHR/IRSC
**Competition/Concours:**	2019-01-24 Team Grant: Transitions in Care/Subvention d'équipe : Transitions dans les soins
**Committee/Comité:**	Team Grant: Transitions in Care/Subvention d'équipe : Transitions dans les soins
**Title/Titre:**	The Digital Bridge: Using technology to support patient-centred transitions from hospital to home

Assessment/Évaluation:

Older adults with multi-morbidity and complex care needs (CCN) are among those most likely to experience frequent care transitions from hospital to home, and many fall into the category of high-cost users, accounting for the majority of year-over-year healthcare spending in Ontario, Canada and internationally. The complexity these individuals stems not only from their multi-morbidity disease profiles, but also the social, environmental and contextual issues that make it difficult for them to manage their physical health needs. The interaction of these challenges often results in frequent hospital admissions. The transition from hospital to home is often challenging leading to poor patient outcomes and higher rates of readmission. Improved communication that enables person-centred care may offer the greatest advantages to support older adults with complex care needs transitioning from hospital to home.

…Krumholz coined the term ‘post-hospital syndrome’ to describe this acquired, transient period of vulnerability post-discharge due to impaired physiological systems and depleted reserves. This depletion limits patients’ ability to adjust and manage their health issues, often leading to hospital re-admission within 30 days with an acute medical illness unrelated to the original diagnosis. Poor communication and incomplete information … can impede access to needed support and resources….

Improving clinician communication is important, the quality and content of that communication with patients also matters. Patients with CCN benefit most from person-centred delivery models that can adapt to their unique needs and engage them as partners in their care. … improve discharge … emphasizing partnership between patient and provider, improving patient self-efficacy, and through improving communication between patients, providers and within care teams. For patients with CCN, incorporating ongoing support for self-care after they return home as part of that communication can offer additional support and benefit. In sum, communication that enables person-centred care and self-management may offer the greatest advantages as we support older adults with CCN transitioning from hospital to home.

The proposal describes the study setting and population information well (e.g. ages 60+ with three or more chronic conditions, English speaking, all hospital providers)

**Review Type/Type d'évaluation:**	Committee Member 3/Membre de comité 3
**Name of Applicant/Nom du chercheur:**	Steele Gray, Carolyn Alice
**Application No./Numéro de demande:**	418448
**Agency/Agence:**	CIHR/IRSC
**Competition/Concours:**	2019-01-24 Team Grant: Transitions in Care/Subvention d'équipe : Transitions dans les soins
**Committee/Comité:**	Team Grant: Transitions in Care/Subvention d'équipe : Transitions dans les soins
**Title/Titre:**	The Digital Bridge: Using technology to support patient-centred transitions from hospital to home

Assessment/Évaluation:

The project will address these three gaps by implementing and evaluating a Digital Bridge to support person-centred health care transitions for older adults with complex care needs. The Digital Bridge will: 1) span organizational and professional boundaries by enabling communication between inter-disciplinary teams working in hospital and primary care, with patients and caregivers; 2) support person-centred care delivery through adoption of co-design methods to establish a workflow; and 3) be evaluated through an implementation science lens.

The Digital Bridge will integrate two tested and validated technologies that are currently in use in hospital and community settings: 1) Care Connector and 2) The electronic Patient Reported Outcomes (ePRO) tool.

Care Connector is an inter-professional communication and collaboration platform initially designed in the hospital setting to support clinical teams caring for patients with CCN. The tool includes discharge communication supports like Patient Oriented Discharge Summaries (PODS), to support clinician communication and collaboration in the community and across care settings. Inter-professional care planner, secured asynchronous messaging, fits clinician workflow. Discharge module. Does not provide ongoing support to patients after transition.

The ePRO tool is a primary care facing technology, iteratively co-designed with patients with CCN, tested and evaluated with patients with CCN, CGs and enable communication on patient-oriented goals, including after transition. The ePRO tool includes two features: 1) My Goal Tracker and 2) Health Journal. My Goal Tracker allows patients and providers to collaboratively create goal-oriented patient care plans and track outcomes related to their goals using a mobile device. SMART (Specified- Measureable-Attainable-Realistic-Time Specific) goal principles guide goal set-up, and Goal-Attainment Scaling is used to measure outcome, argued to be the most appropriate standardized outcome measure for older adults with CCN. Health Journal helps patients, their caregivers and PCPs monitor symptoms and

**Review Type/Type d'évaluation:**	Committee Member 3/Membre de comité 3
**Name of Applicant/Nom du chercheur:**	Steele Gray, Carolyn Alice
**Application No./Numéro de demande:**	418448
**Agency/Agence:**	CIHR/IRSC
**Competition/Concours:**	2019-01-24 Team Grant: Transitions in Care/Subvention d'équipe : Transitions dans les soins
**Committee/Comité:**	Team Grant: Transitions in Care/Subvention d'équipe : Transitions dans les soins
**Title/Titre:**	The Digital Bridge: Using technology to support patient-centred transitions from hospital to home

Assessment/Évaluation:

outcomes most relevant to this patient group. Patients expressed improved collaboration and person-centred care.

The Research Team hypothesize that these two technologies will work synergistically by both supporting the communication and collaboration needs of clinicians and patients at the critical time of care transitions (Care Connector) and engaging patients to set goals and monitor their progress with clinicians starting in the hospital and through their transition back into the community over the longer term (ePRO).

Digital Bridge is an integration of the Care Connector and ePRO technologies that will support care transitions by: 1) inviting PCPs to access Care Connector while the patient is in hospital, allowing for asynchronous communication via the messaging feature for proactive discharge planning, 2) facilitating the inclusion of inter-professional recommendations in the discharge module (e.g. diet and mobility) typically missing from traditional physician generated discharge summaries, 3) electronic generation of PODS for use in patient-centred discharge teaching, 4) providing patients electronic access to PODS post-discharge to facilitate use of information at home, 5) digitally enabling a goal-oriented process to engage patients and families in discharge using ePRO, and 6) providing ongoing self-management support for patients using ePRO for the vulnerable period 6 months post-discharge.

Research Questions (RQ) This project poses three overarching research questions aimed at adapting the technology to local contexts (RQ1), evaluating the impact of the Digital Bridge (RQ2) and exploring the potential to scale and spread the technology (RQ 3). 1. What are the workflow design considerations in adopting digital solutions that bridge care settings to support transitions from hospital to home for patients with CCN, from patient/caregiver, clinician, and organizational perspectives? 2. Is the digital solution a cost-effective means of supporting care transitions to achieve improved processes (improved communication around transitions), patient experience with transitions (improved person-centred care transitions), and patient-reported outcomes (health-related quality of life)? 3. What are the implementation enablers and barriers to adopting technology in this process from patient, caregiver, provider, organizational and system perspectives?

Review Type/Type d'évaluation: Committee Member 3/Membre de comité 3
Name of Applicant/Nom du chercheur: Steele Gray, Carolyn Alice
Application No./Numéro de demande: 418448
Agency/Agence: CIHR/IRSC
Competition/Concours: 2019-01-24 Team Grant: Transitions in Care/Subvention d'équipe : Transitions dans les soins
Committee/Comité: Team Grant: Transitions in Care/Subvention d'équipe : Transitions dans les soins
Title/Titre: The Digital Bridge: Using technology to support patient-centred transitions from hospital to home

---

Assessment/Évaluation:

Digital Bridge will support improved communication amongst the entire care team including providers in the hospital, community, and the patient and family caregivers leading to greater shared situational awareness during care transitions. With wider adoption of the proposed intervention The Research Team anticipate this project will have impact at patient, clinician, organizational and health system levels as reflected in evaluation strategy. For patients this means improved experience with transitions and health-related quality of life. Clinicians may experience greater efficiency in their coordination of care efforts, and likely fewer errors and missed information. At the organizational level the Digital Bridge may be a tool to standardize care transition practices across organizational boundaries. For the health system this project will address the growing challenge of transitioning older adults with CCN from hospital to home, offering a technology enabled solution that could reduce unnecessary readmission or emergency department (ED) visits by patients post-discharge, leading to cost savings.

Research questions will be addressed through two phases of work with a third phase dedicated to developing a scale and spread strategy with provincial and national knowledge user partners. In Phase 1 the applicant will work with providers in hospitals and primary care, patients and their families to co-design a transition workflow enabled through Care Connector and ePRO. In Phase 2 the applicant will implement the new co-designed Digital Bridge (technology plus co-designed workflow) at Sinai Health System and Trillium Health Partners in general medicine (acute) and rehabilitation services. The applicant will engage in a pragmatic developmental evaluation of the tool, comparing patients prior to (n=238) and post (n=238) implementation; assessing impact on transition quality (Care Transition Measure), patient outcomes (health-related quality of life), and communication and inter-professional relations (Relational Coordination Tool). Qualitative interviews, focus groups and observations will collect process and context implementation factors to inform analysis and development of the scale and spread strategy in Phase 3.

workflow

Step 1: Patient onboarding, in-hospital care and connecting to primary care

**Review Type/Type d'évaluation:**	Committee Member 3/Membre de comité 3
**Name of Applicant/Nom du chercheur:**	Steele Gray, Carolyn Alice
**Application No./Numéro de demande:**	418448
**Agency/Agence:**	CIHR/IRSC
**Competition/Concours:**	2019-01-24 Team Grant: Transitions in Care/Subvention d'équipe : Transitions dans les soins
**Committee/Comité:**	Team Grant: Transitions in Care/Subvention d'équipe : Transitions dans les soins
**Title/Titre:**	The Digital Bridge: Using technology to support patient-centred transitions from hospital to home

Assessment/Évaluation:

Step 2: Discharge and transition to primary care team – PODS EPRO

Step 3: 7-day follow-up

Step 4: Maintenance in the community setting

Proposed Study, Methods, and Measures

The study includes three phases: baseline data collection and workflow co-design (Phase 1), implementation and evaluation (Phase 2), and knowledge translation including scale and spread planning (Phase 3).

PHASE 1: Collecting pre-intervention (control) data for the evaluation while engaging in co-design research to adapt validated technologies into new contexts - RQs 1 & 2. – codesign with pt/fam, hospital provider and PCP WG, iterative WG sessions. Feasability and usability assessment adpt FIIT framework, cognitive walk through. Post study system usability questionnaire.

PHASE 2: Implementation, economic and developmental evaluation (RQ 2&3) - pragmatic, real-world implementation and developmental evaluation design to support feasibility. A developmental evaluation approach, in which evaluation questions are used to support decision-making and modifications to improve interventions and programs. Iterative modifications, allowances for sufficient time. Before-after study design.

Review Type/Type d'évaluation: Committee Member 3/Membre de comité 3
Name of Applicant/Nom du chercheur: Steele Gray, Carolyn Alice
Application No./Numéro de demande: 418448
Agency/Agence: CIHR/IRSC
Competition/Concours: 2019-01-24 Team Grant: Transitions in Care/Subvention d'équipe : Transitions dans les soins
Committee/Comité: Team Grant: Transitions in Care/Subvention d'équipe : Transitions dans les soins
Title/Titre: The Digital Bridge: Using technology to support patient-centred transitions from hospital to home

---

Assessment/Évaluation:

Sample size calculations and comparator group defined.

Primary outcomes measure Care Transitions (CTM3), measure of quality transitions estimates and sensitivity analysis. Secondary outcomes: days at home, goals achieved, health related quality of life, etc. Team and provider level processes team communication, relational coordination on four domains: frequent, timely, accurate, problem solving, and three relational domains: shared goals, shared knowledge, respect. Will be measured across four clinical groups over time.

Economic costs: health system data from ICES to compare patients who transitioned out the hospital using DB and the control patient group over one year. Admin data will be combined with patient reported costs to provide an estimate of one year societal costs. CG time costs also estimated. The economic cost plan is robust and goes beyond health system spending to incorporate family and societal costs.

Process evaluation includes implementation assessment with a focus on real world application, adoption of the Consolidated Framework for Implementation Research. Will utilize embedded ethnographic comparative case study approaches, interviews, focus groups, review of relevant documents and participant observation.

Phase 3: KT and scale and spread strategy development will include KT AC and products.

**Review Type/Type d'évaluation:**	Committee Member 3/Membre de comité 3
**Name of Applicant/Nom du chercheur:**	Steele Gray, Carolyn Alice
**Application No./Numéro de demande:**	418448
**Agency/Agence:**	CIHR/IRSC
**Competition/Concours:**	2019-01-24 Team Grant: Transitions in Care/Subvention d'équipe : Transitions dans les soins
**Committee/Comité:**	Team Grant: Transitions in Care/Subvention d'équipe : Transitions dans les soins
**Title/Titre:**	The Digital Bridge: Using technology to support patient-centred transitions from hospital to home

Assessment/Évaluation:

Data Analysis Strategy: appropriate for all phases and all RQs

WEAKNESSES

None

Team

STRENGTHS

Excellent multi-disciplinary team with expertise to carry out study. The proposal will appropriately engage patients and end users throughout the process.

Excellent partnership between research and health system and tech firm.

The project is well supported by its institution(s) and the health system and fully supported to conduct the activities (e.

Review Type/Type d'évaluation: Committee Member 3/Membre de comité 3
Name of Applicant/Nom du chercheur: Steele Gray, Carolyn Alice
Application No./Numéro de demande: 418448
Agency/Agence: CIHR/IRSC
Competition/Concours: 2019-01-24 Team Grant: Transitions in Care/Subvention d'équipe : Transitions dans les soins
Committee/Comité: Team Grant: Transitions in Care/Subvention d'équipe : Transitions dans les soins
Title/Titre: The Digital Bridge: Using technology to support patient-centred transitions from hospital to home

---

Assessment/Évaluation:

g. LTRI, Sinai Health System in Toronto, Institute for Better Health, Trillium Health Partners, Canada Health Infoway, QoC Health Inc). See LOS and matching funds from QoC Health Inc.

WEAKNESSES

None

Capacity Building & Environment

STRENGTHS

The research team provides opportunities for several trainees and summer students including a post-doctoral fellow. The management and leadership model consists of two Implementation Teams to support ground technology deployment and provider engagement, and communication, as well as provide knowledge transfer expertise for the business case for scale and spread.

WEAKNESSES

Review Type/Type d'évaluation: Committee Member 3/Membre de comité 3
Name of Applicant/Nom du chercheur: Steele Gray, Carolyn Alice
Application No./Numéro de demande: 418448
Agency/Agence: CIHR/IRSC
Competition/Concours: 2019-01-24 Team Grant: Transitions in Care/Subvention d'équipe : Transitions dans les soins
Committee/Comité: Team Grant: Transitions in Care/Subvention d'équipe : Transitions dans les soins
Title/Titre: The Digital Bridge: Using technology to support patient-centred transitions from hospital to home

---

Assessment/Évaluation:

None

Viability of the eHealth innovation partner(s) (Stream 2 only)

STRENGTHS

The partners are well positioned to deliver an eHealth innovation and scale up of solution. Clear interest in commercialization given the partnership with QoC Health Inc and ongoing research translation activities of the research team.

WEAKNESSES

None

Review Type/Type d'évaluation: Committee Member 4/Membre de comité 4
Name of Applicant/Nom du chercheur: Steele Gray, Carolyn Alice
Application No./Numéro de demande: 418448
Agency/Agence: CIHR/IRSC
Competition/Concours: 2019-01-24 Team Grant: Transitions in Care/Subvention d'équipe : Transitions dans les soins
Committee/Comité: Team Grant: Transitions in Care/Subvention d'équipe : Transitions dans les soins
Title/Titre: The Digital Bridge: Using technology to support patient-centred transitions from hospital to home

---

Assessment/Évaluation:

Competition: **CIHR Team Grant: Transitions In Care 2019**

Project Application Title: **TG418448 - The Digital Bridge: Using technology to support patient-centred transitions from hospital to home**

1. Research Question
a. Research question(s) is/are clearly stated. **- YES**
b. Strong scientific rationale for pursuing the proposed evaluation. **- YES**
c. Extent to which the research project responds to the objectives of the funding opportunity. **- HIGH**

2. Research Approach
a. Strength of the research approach and justification for the proposed methods/strategies that is supported by available evidence and/or literature. **- HIGH**
b. Appropriateness and rigor of the proposed study design to address the research question(s). **- HIGH**
c. Appropriate incorporation and justification of sex as a biological variable and/or gender as a social determinant of health where applicable.**YES**
d. Appropriate incorporation of Indigenous culturally relevant theoretical and conceptual frameworks, and Indigenous culturally appropriate research protocols, including Indigenous methodologies where applicable. **YES**

3. Applicants
a. Strength of the applicants, taking into consideration evidence that there is the appropriate expertise, influence, resources and stakeholders who are appropriately and meaningfully involved. **- MODERATE**
b. Appropriate engagement of the knowledge user(s) responsible for, or involved in decision-making of, the activity being evaluated. **- MODERATE**

4. Feasibility
a. Appropriateness of the budget and the justification for the amount requested, including the required budget for knowledge translation and dissemination activities. **- TBD**
b. Suitability of the environment, including availability and accessibility of personnel and tools, to conduct the proposed activities. **- MODERATE**
c. Probability that the project objectives will be met within the proposed timeline. **- TBD**

5. Impact of Research
a. Strength of the dissemination plan. **- MODERATE**
b. Potential of the proposal to advance knowledge and produce high-quality evidence to inform actionable health system changes to improve care transitions. **– HIGH**

**Review Type/Type d'évaluation:**	Committee Member 4/Membre de comité 4
**Name of Applicant/Nom du chercheur:**	Steele Gray, Carolyn Alice
**Application No./Numéro de demande:**	418448
**Agency/Agence:**	CIHR/IRSC
**Competition/Concours:**	2019-01-24 Team Grant: Transitions in Care/Subvention d'équipe : Transitions dans les soins
**Committee/Comité:**	Team Grant: Transitions in Care/Subvention d'équipe : Transitions dans les soins
**Title/Titre:**	The Digital Bridge: Using technology to support patient-centred transitions from hospital to home

Assessment/Évaluation:

NOTES/COMMENTS: A dizzyingly detailed submission. Sectional page/word count limits may improve future digestibility. Why should use of this proposed e-health innovation be limited only to older adults? Given suitable strength and stability of the tech provider partner, and appropriate tech literacy orientation training for users, this shows strong potential to scale and spread to support a more demographically diverse population. More proportional patient/caregiver representation on the research team would be most preferable.

Review Type/Type d'évaluation: SO Notes /Notes de l'agent scientifique
Name of Applicant/Nom du chercheur: Steele Gray, Carolyn Alice
Application No./Numéro de demande: 418448
Agency/Agence: CIHR/IRSC
Competition/Concours: 2019-01-24 Team Grant: Transitions in Care/Subvention d'équipe : Transitions dans les soins
Committee/Comité: Team Grant: Transitions in Care/Subvention d'équipe : Transitions dans les soins
Title/Titre: The Digital Bridge: Using technology to support patient-centred transitions from hospital to home

---

Assessment/Évaluation:

Competition: 201901TRC

PRC: TRC

NPA: Carolyn Steele Gray

Application Number: 418448

Project Title: The Digital Bridge: Using technology to support patient-centred transitions from hospital to home

SO Notes begin here:

\`\`

Strengths:

This project proposes to develop a digital bridge from hospital to post-discharge phases. Patients and families will co-design the intervention. They will perform a pragmatic evaluation of the tool. The rationale was sound and the study was well aligned with the funding opportunity. The economic evaluation and qualitative studies were well described. The team has a strong track record with relevant expertise, with a viable tech partner. They are well positioned to conduct this project.

Weaknesses:

The project was relatively simple methodologically and was viewed as not ambitious thus having limited potential impact. The full patient medical record was not included in the study, but was deemed to be an essential component of an effective intervention. The pre-post design is weak and susceptible to bias. Measures to be collected before and after implementation were not clear. While missing data are likely to be problematic, strategies for dealing with missing data were not addressed. Real world generalizability considerations should be addressed. Integration of the intervention with discharge planning should be more clearly discussed. Questions were raised about proportional patient representation on the research team.

Review Type/Type d'évaluation: SO Notes /Notes de l'agent scientifique
Name of Applicant/Nom du chercheur: Steele Gray, Carolyn Alice
Application No./Numéro de demande: 418448
Agency/Agence: CIHR/IRSC
Competition/Concours: 2019-01-24 Team Grant: Transitions in Care/Subvention d'équipe : Transitions dans les soins
Committee/Comité: Team Grant: Transitions in Care/Subvention d'équipe : Transitions dans les soins
Title/Titre: The Digital Bridge: Using technology to support patient-centred transitions from hospital to home

---

Assessment/Évaluation:

Budget:

**** Patient reimbursement needs justification ****

````````````````````````````````````````````````````````````````````````````````````````````````````````````````````````````````

***SO Notes end here.***
````````````````````````````````````````````````````````````````````````````````````````````````````````````````````````````````
